# Label-Free Mass Spectrometry Proteomics Reveals Different Pathways Modulated in THP-1 Cells Infected with Therapeutic Failure and Drug Resistance *Leishmania infantum* Clinical Isolates

**DOI:** 10.1021/acsinfecdis.2c00457

**Published:** 2023-02-10

**Authors:** Lorenzo Tagliazucchi, Ana Perea-Martinez, Greta Fiorini, José Ignacio Manzano, Filippo Genovese, Raquel García-Hernández, Diego Pinetti, Francisco Gamarro, Maria Paola Costi

**Affiliations:** † Department of Life Science, University of Modena and Reggio Emilia, Via Campi 103, 41125 Modena, Italy; ‡ Clinical and Experimental Medicine (CEM) Ph.D. Program, University of Modena and Reggio Emilia, Via Campi 287, 41125 Modena, Italy; § Instituto de Parasitología y Biomedicina “López-Neyra” (IPBLN-CSIC), Avda. del Conocimiento 17, 18016 Armilla, Granada, Spain; ∥ Centro Interdipartimentale Grandi Strumenti (CIGS), University of Modena and Reggio Emilia, Via Campi 213/A, 41125 Modena, Italy

**Keywords:** leishmaniasis, human macrophages, proteomics, mass spectrometry, bioinformatics, *Leishmania* drug resistance, *Leishmania* treatment failure

## Abstract

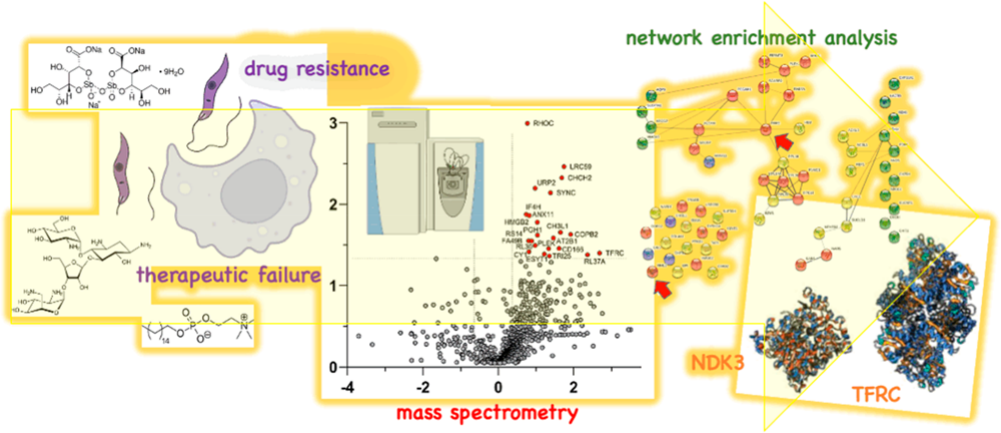

As the world is facing increasing difficulties to treat leishmaniasis with current therapies, deeper investigation into the molecular mechanisms responsible for both drug resistance and treatment failure (TF) is essential in drug discovery and development. So far, few available drugs cause severe side effects and have developed several resistance mechanisms. Drug resistance and TF parasite strains from clinical isolates may have acquired altered expression of proteins that characterize specific mechanisms leading to therapy inefficacy. This work aims to identify the biochemical pathways of THP-1 human monocytes infected by different *Leishmania infantum* clinical isolates from patients with either resistance or with TF outcome, using whole cell differential Mass Spectrometry proteomics. We have adopted network enrichment analysis to integrate the transcriptomics and the proteomic results of infected cells studies. Transferrin receptor C (TFRC) and nucleoside diphosphate kinase 3 (NDK3) were discovered as overexpressed proteins in THP-1 cells infected with paromomycin, antimony, and miltefosine resistant *L. infantum* lines. The overall achievements represent founding concepts to confirm new targets involved in the parasitic drug resistance and TF mechanisms, and to consider in perspective the importance of a dual host–guest pharmacological approach to treat the acute stage of the disease.

Leishmaniasis is a broad-spectrum vector-borne disease spread in tropical and subtropical areas and in the Mediterranean region.^1,2^ It directly affects 98 countries and is responsible for 12 million infections worldwide.^3−5^ The infection is caused by an obligate intracellular protozoa carried by over 30 species of sandfly vectors (*Phlebotomus*) and hits both humans and small mammals like dogs.^6,7^ Because there is scarce economic interest in drug discovery to overcome common resistance mechanisms typical of the available chemotherapeutic agents and the disease hits mainly counties burdened by extreme poverty, the medical and scientific community refers to it as a neglected tropical disease (NTD).^8^ A few pharmaceutical drugs are currently still employed in clinical practice. Liposomal amphotericin B (AmBisome) is preferably used to treat visceral leishmaniasis (VL), together with miltefosine (MIL) in all endemic areas of the disease, as approved by the FDA in 2014.^9,10^ Other older chemotherapeutics include pentamidine, paromomycin, and pentavalent antimony (Sb^V^), such as sodium stibogluconate (Pentostam).^11−13^

The main causes of Leishmaniasis therapeutic failure (TF) with the previously mentioned drugs are due to patients’ unresponsiveness to the drugs because of comorbidity or immunosuppressive conditions (e.g., AIDS) or to the biochemical resistance mechanisms themselves that several guest strains have developed.^14,13^ The former issue relates to unexpected drug reactions in which the expected chemotherapeutic effect does not occur due to the variability of response to the therapeutic schemes and is caused by diverse and co-occurring factors (etiological, pathological, environmental, or genetic).^15,16^ The latter issue falls under the drug resistance mechanisms, often due to genetic mutations that lessen the parasite’s response to a therapeutic protocol when the parasite is under drug pressure.^17^ Drug resistance causes prevalently include molecular modifications at the plasma membrane of the parasite, resulting in decreased drug uptake or increased export/efflux, as well as cytosolic drug inactivation by host metabolism or compartmental sequestration.^17−19^ Likewise, alterations in the levels of the primary target can occur due to decreased target affinity for the drug or complete loss of target as a guest counter-reaction to prolonged drug exposure.^19,20^ The ability of different clinical isolates of *Leishmania infantum* strains to modulate the transcriptome of THP-1 cells, a human acute monocytic leukemia cell line that lacks of surface and cytoplasmic immunoglobulins,^21^ was recently reported by García-Hernández *et al.* and Perea-Martínez *et al.*(22,23)

In their promastigotes morphology, *Leishmania* guests bind to surface receptors on macrophages and monocytic blood cells, and are internalized by receptor-mediated phagocytosis, usually enhanced by specific cytoskeletal proteins.^24^ The initial promastigote-macrophage crosstalk is essential to the establishment of the host infection, as the parasite survival depends on its ability to escape host digestion and avoid protein lysis.^25,26^*Leishmania* parasites have evolved many strategies to deal with the microbicidal power of the immune cells and its host effective immune response and to enhance nutrient uptake by acting on extracellular receptors.^27^ The infected macrophage can metabolize the required Fe^II^, necessary for host survival, and at the same time it minimizes the undesired oxidative properties of the excess cofactor.^27,28^ During acute infection, when the host withdraws iron from the circulation to prevent parasite spread, much crosstalk between the parasite and the host cells has been described to guarantee a continuous metal supply.^29,30^ Indeed, this study assumes that THP-1 and *Leishmania* proteins crosstalk for parasite survival and reproduction and that the host proteome is affected by *Leishmania* ssp. also in the development of drug resistance or in TF events. For this reason, the present study aims to investigate the THP-1 host proteins and biochemical pathways directly involved in *L. infantum* drug resistance and TF mechanisms against the most common guest-directed antiparasitic agents such as miltefosine, paronomycin, and antimonials in order to discover how the human proteome is modulated after infection with different *L. infantum* lines. The emerging functional pathways and the associated single proteins represent a starting point for the development of new therapeutic approaches against leishmaniasis.

During previous studies it was demonstrated for the first time that mass spectrometry (MS) was the highest-performing tool to characterize the *Leishmania*–macrophage interaction at the first stage of their infection,^31^ which was previously accomplished by blotting techniques.^32^ Since then, a few other experiments were set up with MS-based proteomics using either a labeling or label-free approach, and they were all performed to disclose the molecular bases of guest–host crosstalk in different *Leishmania* species without considering drug-resistant lines or TF effects.^33−36^ Our MS approach exploits a similar proteomic analysis of THP-1 host cells infected with TF and drug-resistant *L. infantum* lines from clinical patients, and the analysis focuses on the modulation of the proteome that is directly observed in the events of resistances to the most common therapeutics and TF.

For this purpose, we have set up a whole-cell, label-free MS proteomics investigation on *in vitro* samples of THP-1 cells infected with *Leishmania* clinical isolates characterized by TF (four strains) and elective drug resistance (three strains) to identify the most relevant proteins putatively involved in drug resistance and/or TF after the infection of human host cells. THP-1 total proteomes were characterized by bottom-up LC-MS/MS sequencing and compared to the respective untreated/sensitive controls through ANOVA to evidence the differentially expressed proteins (DEPs). DEPs have undergone biochemical network enrichment analysis using STRING (Protein–Protein Interaction Networks Functional Enrichment Analysis),^37^ and the outcomes were integrated with the outcome of the transcriptomics experiments performed on the same cell lines with gene expression analysis and RNA-seq.^22,23^ Finally, we have mapped the functional interactomes and biological processes with the Gene Ontology (GO) tool^38^ to evidence cellular protein dysregulation due to resistance onset phenomena. The experimental workflow is illustrated in Figure 1.

**Figure 1:**
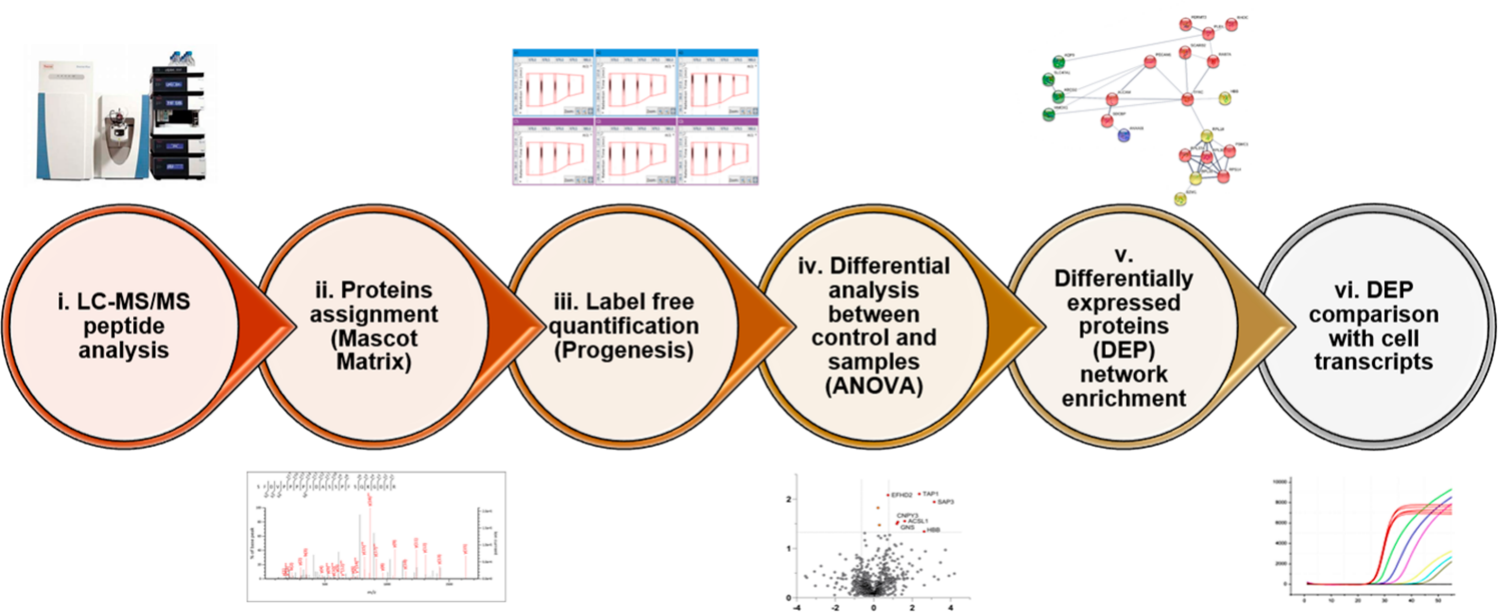
Analytic workflow from LC-MS/MS runs to comparison of differentially expressed proteins (DEPs) with mRNA transcripts. Digested, desalted peptides from sample lysates were injected into LC-MS/MS in data-dependent acquisition (DDA). Raw files were processed with the Mascot suite (qualitatively) and Progenesis QI for Proteomics (quantitatively) for label-free quantitation analysis (one-way ANOVA test). Emerging DEPs for each comparison group vs control were studied through network enrichment analysis (STRING) and integrated with differentially expressed transcripts (DETs) of the same line from García-Hernández *et al.*(22) and Perea-Martínez *et al.*(23)

In this optic, we propose new target pathways and proteins that can represent the basis for an innovative anti-infective approach known as host-directed therapy (HDT), which aims at depriving the parasite of host pathways exploited for its persistence in a hostile environment.^39^ Indeed, targeting the host and the parasite in the same time frame might be a straightforward strategy to reduce toxicity and resistance by considering *ad hoc* drug combinations and/or designing chimera molecules.^40^

## Results and Discussion

### Mass Spectrometry Proteomics

The liquid chromatography tandem mass spectrometry (LC-MS/MS) analysis was performed on the immortalized monocyte-like cell line THP-1 infected with different strains of *L. infantum* previously described by García-Hernández *et al.*(22) and Perea-Martinez *et al.*(23) Three promastigote *Leishmania* lines were used to represent drug resistance toward paromomycin (Hi-L2126), MIL (Hi-L5159), and Sb^III^ (Hi-L3323) along with four nonresistant lines (Hi-L2165, Hi-L2221, Hi-L2070, and Hi-L2255), which were isolated from VL patients with a TF outcome in polytherapy regimes with traditional chemotherapeutics.^13,22^ In parallel, a negative (noninfected) THP-1 control (Hi-LJPC) was grown along with a positive sample, which was obtained through parasite exposure after a heat-inactivation cycle (Hi-L death). These samples were provided to eliminate the proteins associated with phagolysosome processes and/or vesicular trafficking and not specifically related to drug resistance or TF pathways.^22,23^ Information about the promastigote cell line features is provided in Table S1. A typical bottom-up digestion protocol was applied to all of the samples before analysis with an Orbitrap Q-Exactive mass spectrometer (Thermo Fisher). Proteins were identified and quantified with the Mascot Matrix suite and the Progenesis QI suite (Nonlinear Dynamics, Waters Corporation). Details on sample preparation and LC-MS/MS analysis are provided in Methods and Tables S1, S2, and S3. Specifications about peptide identification are provided in Methods and Tables S4 and S5.

**Figure 2:**
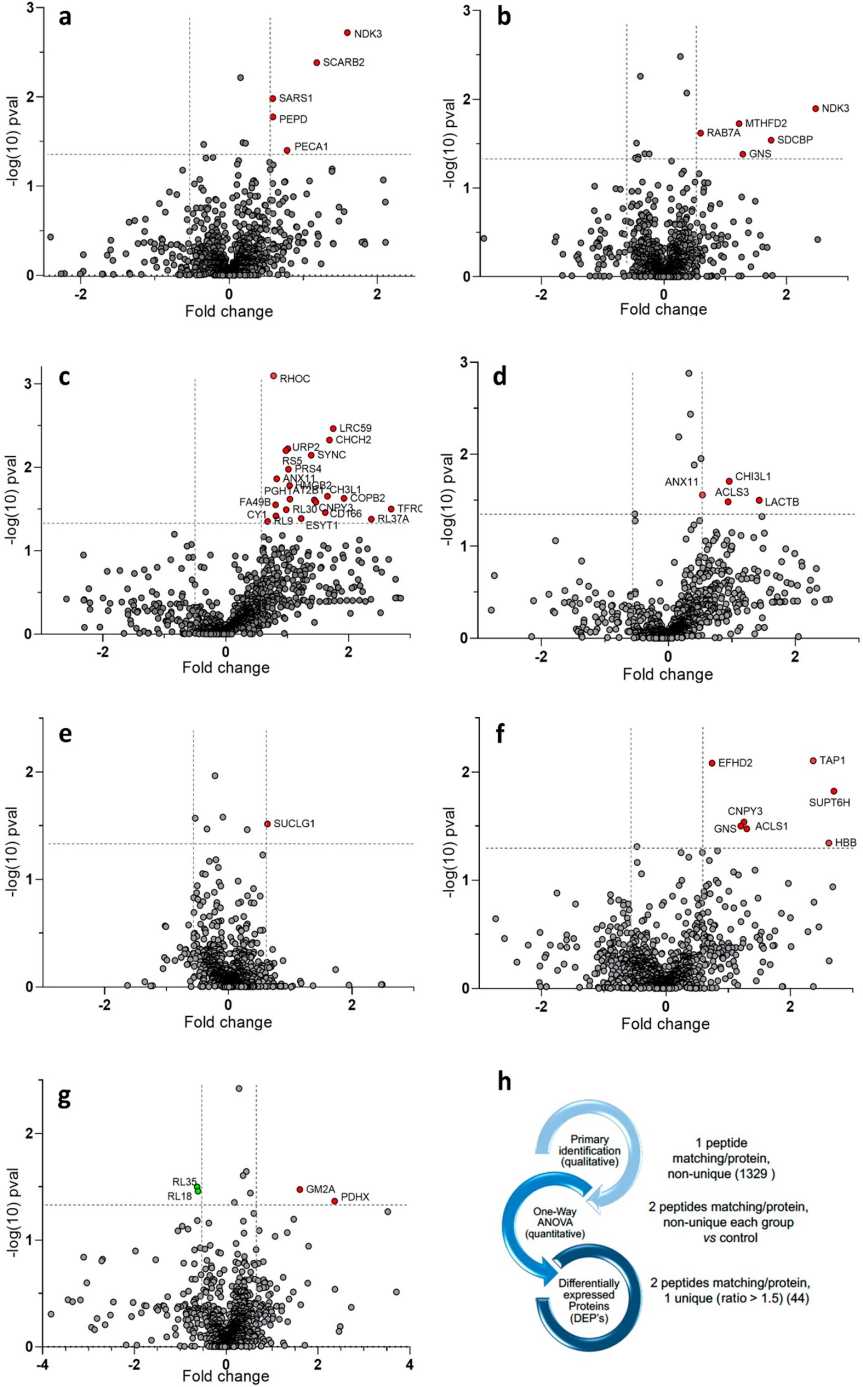
(a–g) Volcano plots representing the differential analysis between the control group and the infected cell lines. (a–c) THP-1 cells infected with drug-resistant lines: (a) Hi-L3323, (b) Hi-L2126, and (c) Hi-L5159. (d–g) THP-1 cells infected with TF lines: (d) Hi-L2070, (e) Hi-L2165, (f) Hi-L2221, and (g) Hi-L2255. The plots show log_10_(*p* value) associated with proteins on the *Y* axis vs the fold change [FC = log_2_(ratio)] on the *X* axis. Red dots refer to proteins significantly (*p* value < 0.05) differentially upregulated (ratio ≥ 1.5, FC ≥ 0.58) compared to the infected group. Green dots refer to proteins significantly (*p* value < 0.05) differentially downregulated (ratio ≤ 1.5^–1^, FC ≤ −0.58) with respect to the control group. Comparative lists are provided in Table S6. (h) Peptide matching and protein refinement workflow.

Overall, we identified a total of 1329 human proteins before quantification, with at least one nonunique peptide for protein matching. We performed the quantitative analysis in the comparison between each strain and its reference by imposing the criteria of at least two peptides/one unique for protein matching. Each differential test identified a different number of proteins for every strain pair.^41^ In the case of THP-1 cells infected with drug-resistant *L. infantum* lines, 1063 proteins were detected by the test between Hi-L3323 samples and Hi-LJPC considered as a negative control (973 with at least one unique peptide), 1058 proteins in Hi-L2126 samples versus control (951 with at least one unique peptide), and 1080 proteins in Hi-L5159 samples versus control (981 with at least one unique peptide). When the analysis was focused on THP-1 cells infected with TF *L. infantum* lines versus control, we identified 932 proteins in Hi-L2165 samples (871 with at least one unique peptide), 961 proteins in Hi-L2221 samples (897 with at least one unique peptide), 1070 proteins in Hi-L2070 samples (968 with at least one unique peptide) and 1016 proteins in Hi-L2255 samples (926 with at least one unique peptide). Details on protein accessions are provided in Table S6. Proteins were matched with the SwissProt database^42^ on the MascotMatrix ion search suite,^43^ with a *p* value corrected to a 1% false discovery rate (FDR). Forty-four differentially expressed proteins (DEPs) emerged from a comparison between the control group and each infected cell line as described before (*p* value < 0.05, ratio ≥ 1.5), represented by the volcano plots [log_10_(*p* value) vs fold change (FC = log_2_(ratio))] in Figure 2.

All of the samples underwent exclusion of nonspecific DEPs. For this purpose, preliminary one-way ANOVA was applied between the positive control (THP-1 transfected with heat-inactivated parasites) and negative control (blank, THP-1 host without *Leishmania* parasite), and the resulting DEPs (*p* value < 0.05, ratio ≥ 1.5) were excluded from the other samples outcome. These proteins are likely to represent biological processes associated with general parasite infection, mainly related to phagolysosome-mediated uptake processes and not directly to resistance phenomena.^36,41,44^

**Figure 3:**
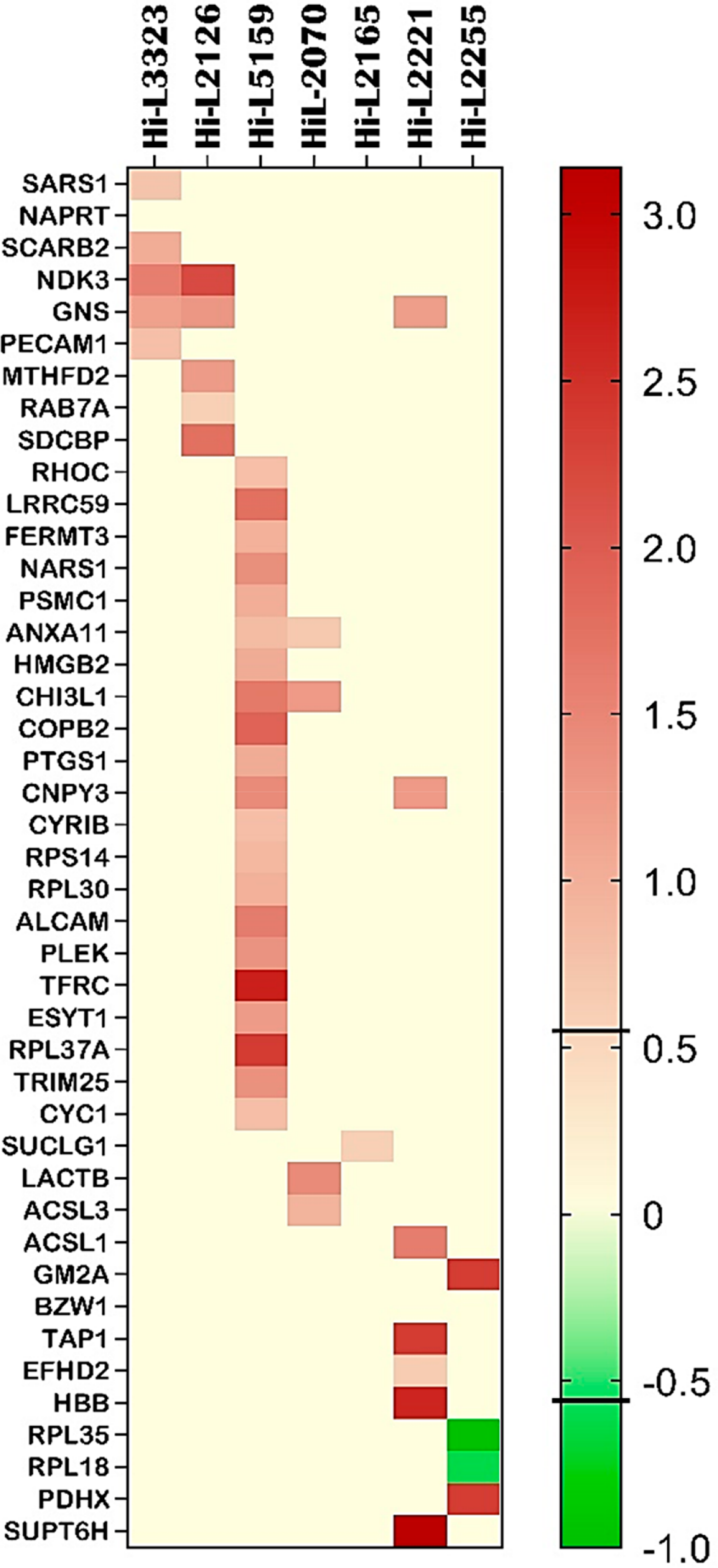
Heat map of the differentially expressed proteins that emerged from the one-way ANOVA test between treated lines and control groups of all strains studied. From left to right, lines HiL-3323, HiL-2126, and HiL-5159 are drug-resistant, and the others belong to the TF group. Proteins with *p* value < 0.05 and FC ≥ 0.58 (ratio ≥ 1.5) for upregulation or FC ≤ −0.58 (ratio ≤ 1.5^–1^) for downregulation were considered DEPs. Upregulated proteins are represented as dark-brown cells, and downregulated proteins are represented in green. Most of the listed proteins did not display any significant FC and are represented in pale yellow. The bar on the right side represents the FC legend. The heat map was obtained using GraphPad Prism 9.3.1 (accessed in January 2022).^45^ Raw data from the heat map are reported in Table S7.

The differential ANOVA test evidenced 44 DEPs. They include some significant proteins like the mitochondrial X component of pyruvate dehydrogenase complex (PDHX), the mitochondrial bifunctional methylenetetrahydrofolate dehydrogenase/cyclohydrolase (MTHFD2), and *N*-acetylglucosamine-6-sulfatase (GNS) as upregulated proteins (ratio ≥ 1.5, *p* value < 0.05) and 60S ribosomal proteins L18 and L35 (RPL18 and RPL35) as downregulated proteins (ratio ≤ 1.5^–1^, *p* value < 0.05). Figure 3 shows the ANOVA results in a heat map format. The table with all identified proteins is reported in Supporting Information 2.

### Network Enrichment Analysis and Biological Process Identification

**Figure 4:**
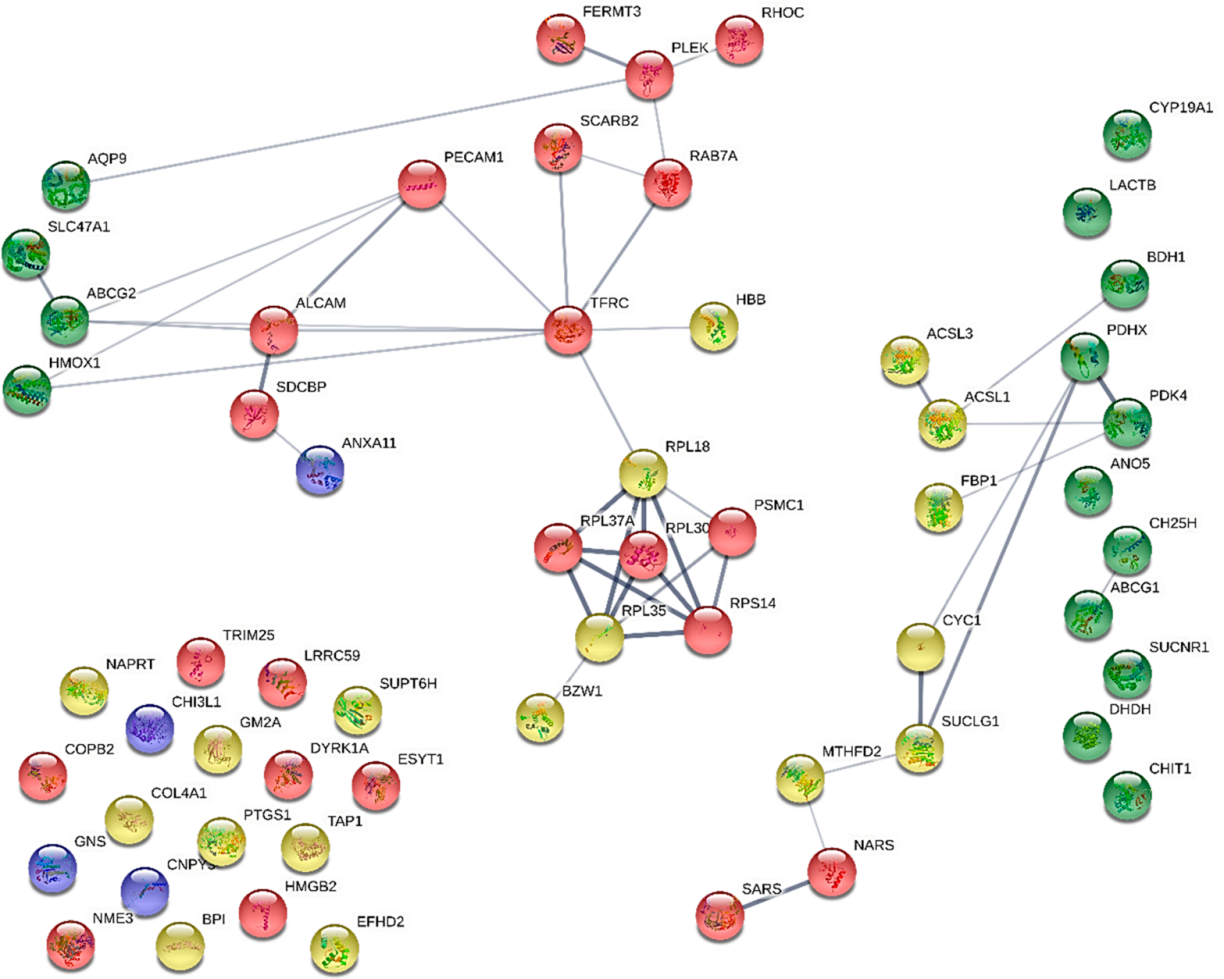
First-level interacting network analysis obtained with STRING. The interconnections among 60 proteins/transcripts as input (44 DEPs and 18 DETs) are shown without further enrichment. Red spheres represent DEPs from drug-resistant lines. Yellow spheres represent DEPs from TF lines. Blue spheres represent DEPs from both drug-resistant and TF lines. Proteins corresponding to transcriptomic output^22,23^ are colored in green and lie vertically on the left and the right sides of the network. STRING was used with basic settings, in which the edges indicate both functional and physical protein associations; the meaning of network edges is confidence (line thickness indicates the strength of data support); the active interaction sources are experiments, databases, coexpression, neighborhood, gene fusion, and co-occurrence; the minimum required interaction score is medium confidence (0.400); the maximum number of interactions to show is first shell (no more than 10 interactors) and second shell (custom value, 60 interactors maximum). Network statistics: the number of nodes is 60; the number of edges is 46; the average node degree is 1.53; the average local clustering coefficient is 0.395; the expected number of edges is 29; the PPI enrichment *p* value is 0.00215 (https://string-db.org/; last accessed January 21, 2022). In the network all proteins are reported with their gene code. NKD3 is reported as NME3 (see Table S6).

The transcriptomic research previously applied to the same sample of the present proteomic analysis identified 18 differentially expressed transcripts (DETs) among the seven cell lines analyzed, which represent the transcripts characterizing the drug-resistant and TF cell lines (Table S8).^17,18^ A comparison between the DEPs and the DETs was run before and after a network enrichment process, adopting the statistical methods provided in Methods. To physically represent the direct outcome of the first level of the enrichment process, all the proteomic data, including both DEPs (44) and DETs (18) matching the imposed criteria, were entered as UniProt^46^ accessions to the STRING database, allowing a first-level approach to protein–protein interaction (PPI) network functional enrichment analysis.^37^ The network was generated according to the STRING criteria by adopting curated databases, known interactions experimentally determined, gene fusion, and co-occurrence (Figure 4). The first level of the network enrichment analysis represented 60 proteins/transcripts belonging to either proteomics or transcriptomics experimental datasets. The interactome reported in Figure 4 revealed the overlap of two mutual proteins/transcripts as the outcome of the direct STRING network enrichment (confidence < 0.400): TFRC, overexpressed in THP-1 cells infected with the MIL-resistant line (Hi-L5159), and NKD3, overexpressed in THP-1 cells infected with paromomycin- and Sb^III^-resistant lines (Hi-L3323 and Hi-L2126, respectively), referring specifically to drug-resistant lines and not present in TF lines. The former protein is a cell surface receptor necessary for cellular iron uptake by the process of receptor-mediated endocytosis. The latter plays a major role in the synthesis of nucleoside triphosphates other than ATP through a phosphorylated active-site intermediate.^47^ In addition to the two overlapping proteins/transcripts, TFRC and NDK3, some proteins from the two different datasets appear to belong to mutual pathways and interact with each other. Remarkable examples are the subnetworks consisting of PDHX, PDK4, CYC1, BHD1, ACSL1, and ACSL3, involved in biosynthetic processes of fatty acid derivatives, purine ribonucleotides, and the tricarboxylic acid cycle,^48,49^ that are shared among the DEP and DET datasets. Subsequently, ABCG2 and HMOX, networking with TFRC, ALCAM and PECAM, and AQP9 and PLEK, participate in immune response and the immune system and in vesicle-mediated trafficking.^50^ Also, the presence of some emerging metabolic networks (islands/clusters) is outstanding, the most prominent of which includes subunit proteins of the ribosomal complex (RPL37A, RPL30, RPS14, RPL18, RPL35, PSMC1, and BZW1) of the host, as was already observed by other authors.^51^ Details on the analyzed proteins, transcripts, and their database accessions are provided in Table S6. This analysis points out a strong relationship between proteomics and transcriptomics and emphasizes the role of certain biological pathways that emerge at the first level of the enrichment process.

**Figure 5:**
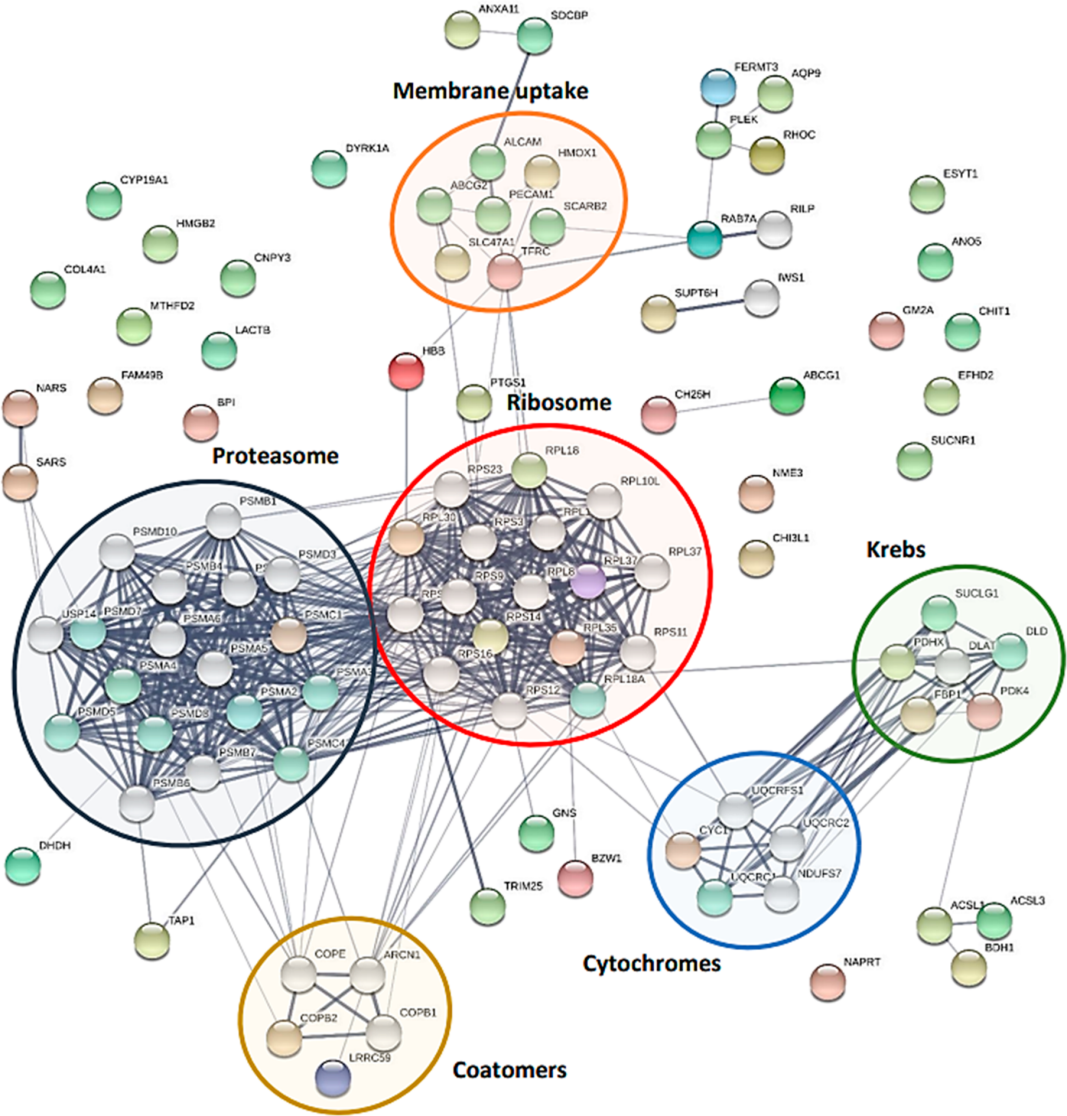
Most representative biochemical pathways (islands) that emerged in STRING enriched networks. Each colored circle represents a different system, as labeled. The number of nodes is 100. The number of edges is 505. The average node degree is 10.1. The average local clustering coefficient is 0.618. The expected number of edges is 189. The PPI enrichment *p* value is <1.0 × 10^–16^ (https://string-db.org/, last accessed on February 20, 2022). Information about the proteins in the network and their GO annotations are provided in Table S9.

Afterward, progressive PPI enrichment processes were performed with STRING using the network statistic specified in Methods, as reported in Figure 5. The final elaboration led to the identification of several islands comprising different biochemical pathways: iron and hemostasis (connected with the TFRC gene), Krebs cycle and oxidative phosphorylation, cell adhesion, proteasome, ribosome, sulfatases, hydrolases, and coatomers (Figure 5). Even though the transcriptome does not exhibit the exact correspondence of proteins, we have discovered DEPs that are strongly connected with the transcripts because they belong to shared pathways. In the network, the ribosome and proteasome islands stand out as the most populated networks (Figure 5).

**Figure 6:**
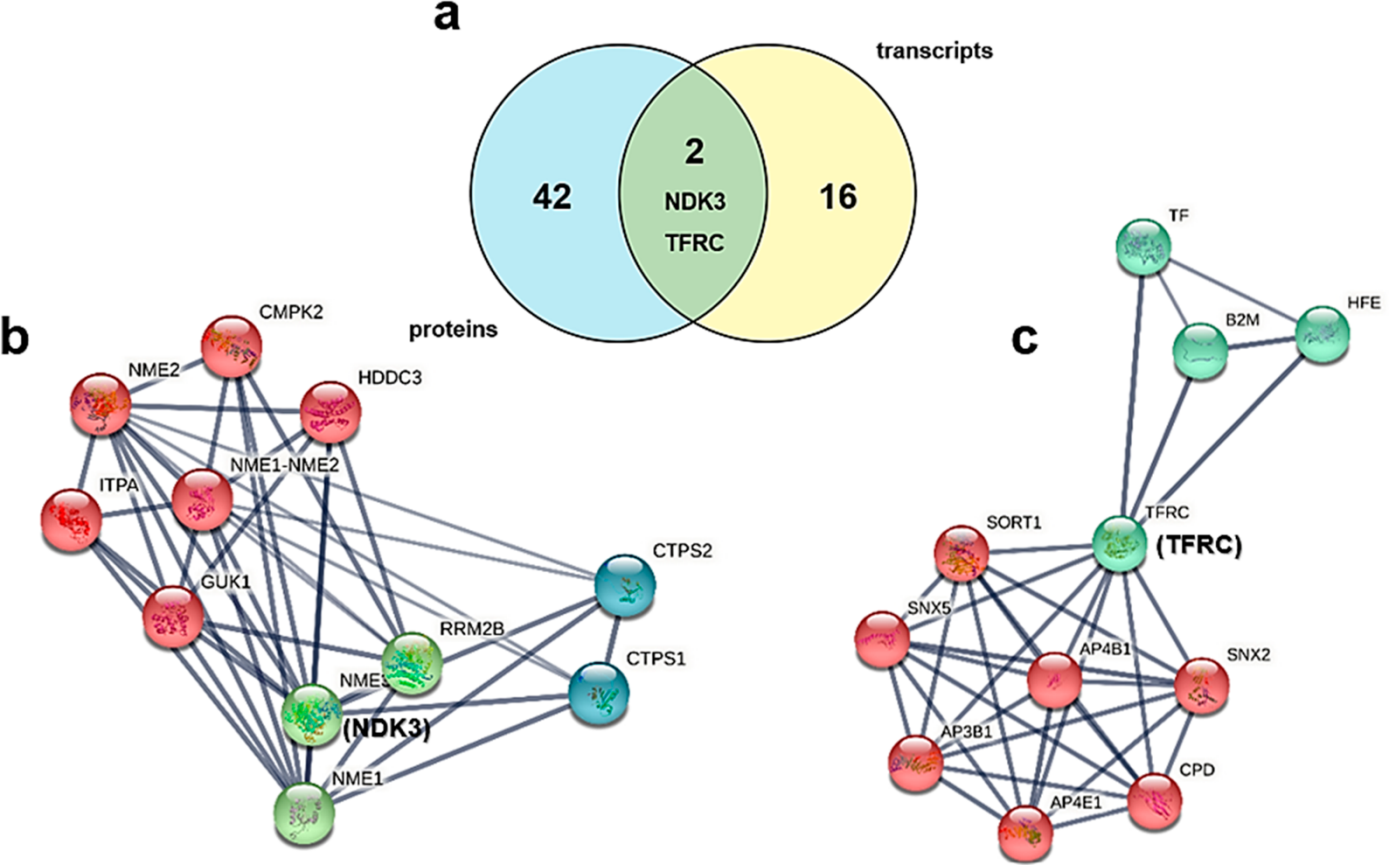
(a) Venn diagram obtained on the Bioinformatics & Evolutionary Genomics website,^54^ showing the proteins overlapping between the two datasets. LC-MS/MS proteomics evidenced 44 differentially expressed proteins, whereas 18 differentially expressed transcripts emerged from mRNA examination.^22,23^ Two proteins/genes are mutual: transferrin receptor 1 (TFRC) and nucleoside diphosphate kinase (NKD3, reported as NME3). (b, c) Main outstanding protein clusters from local network enrichment analysis with STRING of NDK3 and TFRC. Both networks were generated with *k*-means clustering using three cluster inputs (NDK3) or two cluster inputs (TFRC). (b) NKD3 network. The number of nodes is 11, and the number of edges is 40. The average node degree is 7.27. The average local clustering coefficient is 0.861. The expected number of edges is 10. The PPI enrichment *p* value is 2.6 × 10^–12^. The red cluster is associated mainly with nucleotide phosphorylation (GO:0046939). The blue cluster is associated with *de novo* CTP biosynthesis (GO:0044210). The green cluster is associated with the UTP biosynthetic process (GO:0006228). (c) TFRC network. The number of nodes is 11, and the number of edges is 34. The average node degree is 6.18. The average local clustering coefficient is 0.958. The expected number of edges is 11. The PPI enrichment *p* value is 7.25 × 10^–9^. The blue cluster is associated mainly with iron ion transport (GO:0006826). The red cluster is associated with intracellular protein transport (GO:0006886).

The Venn diagram presented in Figure 6a shows how TFRC and NDK3 are the only two proteins belonging to both datasets. The STRING generation of two local networks around NDK3 and TFRC evidences the proteins that directly interact at close distance with the two hit proteins. The NDK3 enriched network (Figure 6b) reporting 11 biomolecules (nodes) evidences the presence of many proteins associated with nucleoside phosphorylation (GO:0046939) and *de novo* CTP biosynthesis (GO:0044210). NDK3 is involved in the *de novo* synthesis of nucleoside triphosphate kinase, transferring its γ-phosphate to the β-position of the nucleoside diphosphate via a ping-pong mechanism. It also has roles in normal hematopoiesis.^52,53^ NKD3 covers also a major role in the trypanosomatid protozoan’s purine metabolism, and it is phosphorylated in antimony-resistant lines. The TFRC network (Figure 6c) displays the interaction of the 11 nearest proteins around TFRC. Two GOs are evidenced in the enrichment: the intracellular protein transport functional network and the iron transport network. TFRC is involved in cellular uptake of iron that occurs via receptor-mediated endocytosis of ligand-occupied transferrin receptors into specialized endosomes, followed by endosomal acidification that allows iron release.^29,30,55^ TFRC is also necessary for the development of erythrocytes and the nervous system.^56^ The parasite employs the host macrophage iron intake for its intracellular growth, resulting in TFRC protein upmodulation.

### Biological Pathway Analysis

Based on the network in Figure 5, 532 GO unique biological process annotations were obtained from the proteomic data, and these have been compared with the 78 GO annotations obtained for transcripts. The GOs were retrieved from STRING analysis and compared with the lowest *p* value (*p* < 0.05). From the comparison, 14 proteins and 11 transcripts emerged as involved in 10 GO biological processes, mutual among proteins and transcripts reported in Table 1.

**Table 1:** The 10 GO Biological Processes of the Proteins Dataset and Transcripts Dataset

protein	transcript(s)^22^	shared GO	qualified GO term
CHI3L1	DHDH	GO:0005975	carbohydrate metabolic process
CHI3L1	CHIT1	GO:0006032	chitin catabolic process
ACSL3	CH25H	GO:0006629	lipid metabolic process
RAB7A	CH25H	GO:0006629	lipid metabolic process
PTGS1	CH25H	GO:0006629	lipid metabolic process
LACTB	CH25H	GO:0006629	lipid metabolic process
ACSL1	CH25H	GO:0006629	lipid metabolic process
GM2A	CH25H	GO:0006629	lipid metabolic process
TFRC	TFRC	GO:0006826	iron ion transport
TFRC	TFRC, HMOX1	GO:0006879	cellular iron ion homeostasis
NDK3	NME3	GO:0006915	apoptotic process
CHI3L1	NME3	GO:0006915	apoptotic process
PECAM1	COL4A1	GO:0030198	extracellular matrix organization
TFRC	FBP1	GO:0035690	cellular response to drug
HMGB2	BPI	GO:0050829	defense response to Gram-negative bacterium
RPL30	BPI	GO:0050829	defense response to Gram-negative bacterium
PSMC1	ABCG2, SLC47A1, ANO5	GO:0055085	transmembrane transport
TAP1	ABCG2, SLC47A1, ANO5	GO:0055085	transmembrane transport

**Figure 7:**
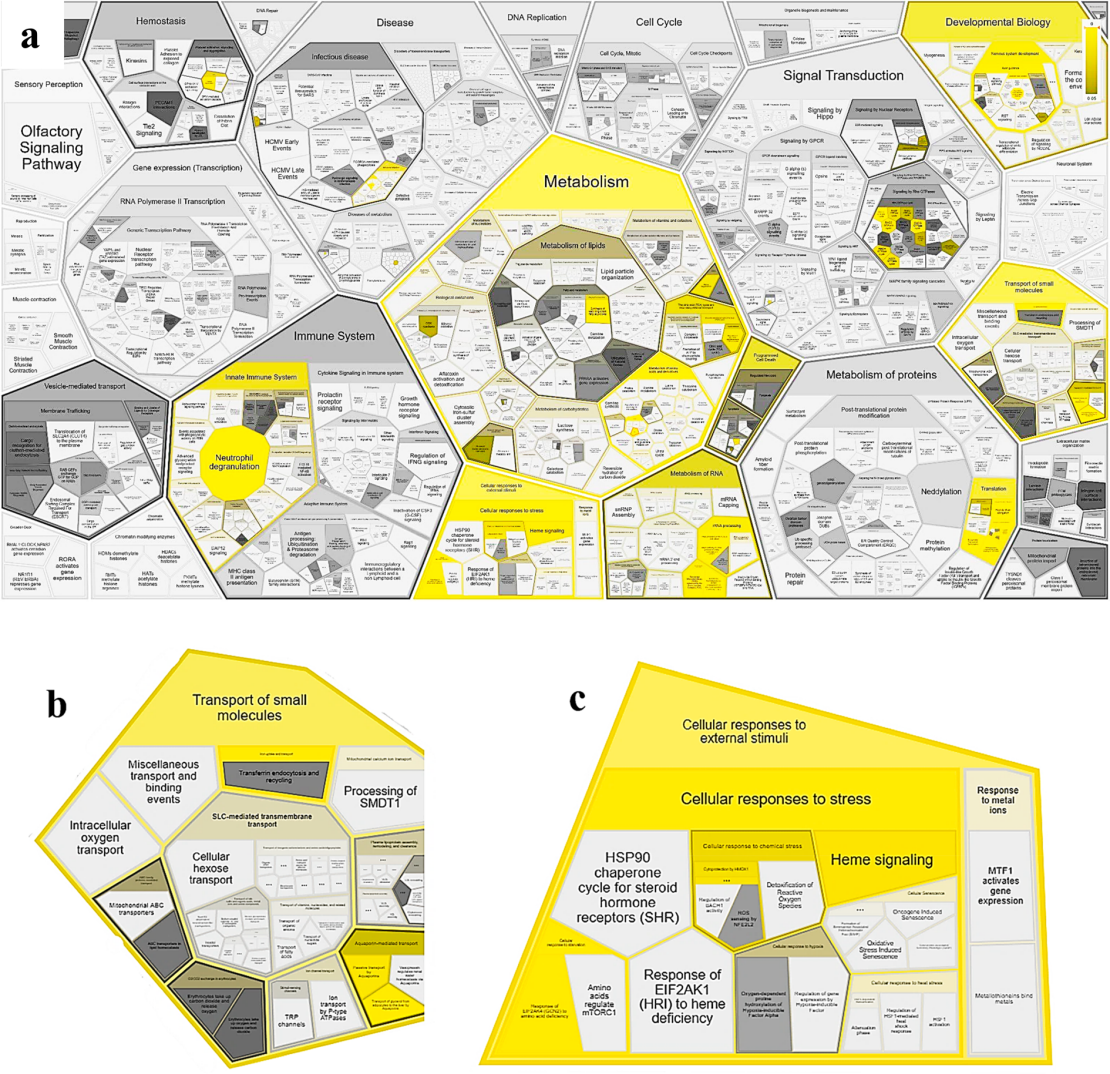
(a) REACTOME Voronoi diagram showing the pathway topology of the 60 proteins/transcripts entered into the tool. This diagram, also known as tessellation, leads to an interactive overview of pathway analysis results, showing in yellow the metabolic pathways in which the entered proteins are involved. The intensity of the yellow color displays the *p* value of the statistical test for overrepresentation: brighter color indicates stronger known involvement of the protein in a certain biological pathway. Shapes filled with dark gray represent pathways without significant overrepresentation, while pathways without assigned proteins are colored in light gray.^62^ The pathway display is tessellated into contiguous regions, each corresponding to a pathway and grouped according to the relationships among pathways specified in the event hierarchy.^63^ (b) Enlargement of (a) displaying the involvement in cellular transport, from small molecules to oxygen. (c) Enlargement of (a) displaying the involvement in the cellular response to external stimuli and stress and in signaling.

From the GO annotations, the iron transport (GO:0006826 and GO:0006879) and the apoptotic process (GO:0006915) emerge clearly, confirming the role of TFRC and NDK3 in leishmaniasis. Also, other biological processes were taken into consideration, and a deeper investigation into GO annotations was performed. Many of the discovered pathways are involved in the persistence of the parasite inside the host: some important pathways are linked to lipid (GO:0006629) and carbohydrate (GO:0005975 and GO:0006032) metabolic processes; others are involved in extracellular matrix organization (GO:0030198 and GO:0055085) and cellular response to drugs (GO:0035690 and GO:0050829). Carbohydrate- and lipid-related processes, although generic, are involved with the supply of energy to the parasite. Other processes show on one hand that the parasite has a degree of influence on transmembrane transport processes exploited to enter the host and on the other hand that certain proteins are overexpressed in response to drug administration; the latter show that the parasite puts in place defense mechanisms to resist within the host even under hostile conditions. The data retrieved from the dataset comparison reported in Table 1 were validated on PANTHER^57,58^ and REACTOME,^59,60^ and four overlapping pathways were isolated and characterized: iron ion transport (GO:0006826, TFRC), cellular iron ion homeostasis (GO:0006879, TFRC); extracellular matrix organization (GO:0030198, COL4A1 and PECAM1), and transmembrane transport (GO:0055085, TAP1).^41^ On the REACTOME analysis tool it was possible to observe where the various selected pathways are located within the metabolism thanks to a Voronoi diagram showing the pathway topology of proteins (Figure 7).^61^

### Protein Set of Reference for RT-qPCR Experiments

A small set of proteins was selected for RT- qPCR validation experiments. A metadata search based on the specificity of the proteins/transcripts reported in the heat map (Figure 3) and Table 1 was performed first, considering their role in physiological metabolism and their involvement in *Leishmania* infection and parasite resistance events or the host’s failure against the treatment. TFRC and NDK3 were identified because they fit the requirements belonging to the drug-resistant cell lines group and are shared among proteomics and transcriptomics. Then two proteins specific for TF, TAP1 and ACSL1, were selected because these are present both in the heat map (Figure 3) with high FC and belong to GOs shared between DEPs and DETs (Table 1) showing transporters and lipid metabolism activity. Another protein, GM2A, which is involved in lipid metabolism, shows a high FC and TF specificity and therefore was included in the panel. An additional protein identified was PDHX, specific for TF (Figure 3), having FC ≥ 2.3 and belonging to the mitochondria metabolism network, an important network that emerged from STRING analysis (Figure 4). Each selected protein was searched on GeneCards using as input six proteins from Table 1 (ACSL1, TAP1, TFRC, NDK3, GM2A, and PDHX). A literature search was performed on these proteins, and their cellular roles and involvement in leishmaniasis are reported in Table 2.

**Table 2:** Descriptions of the Six Proteins That Matched the Imposed Criteria and Were Chosen to Be Investigated in the Official Literature

ACSL1	Acyl-CoA synthetase 1 (ACS1), or long-chain-fatty-acid-CoA ligase 1, is an isozyme of the long-chain fatty-acid-coenzyme A ligase family. ACSL1 activates free long-chain fatty acids, coming from an exogenous or endogenous source, which is the first reaction of their metabolism to fatty acyl-CoA esters, and this activation is required for both synthesis of cellular lipids as part of anabolic lipid metabolism and their degradation via β-oxidation as part of catabolic lipid metabolism. The carbohydrate and lipid metabolisms were the most altered pathways in response to *Leishmania* infection, as the parasite could exploit the host’s organelles to obtain energy.^64^ Although not strictly related to drug resistance, due to its metabolic relevance it has gained interest as a drug target for antileishmanial therapies.^65^
TAP1	Transporter associated with antigen processing 1 is also known as transporter 1, ATP binding cassette subfamily B member. TAP1 is a member of the superfamily of ATP-binding cassette (ABC) transporters; therefore, it is additionally denoted as ABCB2. The ABC transporters are also associated with antigen processing (TAP) and adaptive immunity. TAP1 seems to have an important role in antigen presenting in parasitic diseases, including *Leishmania major* and *Toxoplasma gondii*.^66,67^
NDK3	Members of the nucleoside diphosphate kinase (NDK) family are ubiquitous and reversibly convert nucleoside diphosphates to the corresponding nucleoside triphosphates by transferring to the former the phosphate in the γ-position from another nucleoside triphosphate. The reaction is Mg^2+^-dependent and proceeds by a ping-pong mechanism that involves an intermediate activated state containing a phosphorylated histidine. NDK enzymes play a fundamental role in *Leishmania* infection since the parasite lacks the ability to synthesize purine nucleotides *de novo* and takes advantage of its host’s enzymes to survive and proliferate. Its overexpression relates to the parasite’s need for nucleoside supply.^68^
GM2A	Ganglioside GM2 activator protein is a liposomal protein that catalyzes degradation of glycosphingolipids with terminal α-galactosyl residues in most non-neuronal tissues and in body fluids. The large binding pocket can accommodate several single-chain phospholipids and fatty acids, and GM2A also exhibits some calcium-independent phospholipase activity. It binds gangliosides and stimulates ganglioside GM2 degradation. It stimulates only the breakdown of ganglioside GM2 and glycolipid GA2 by β-hexosaminidase A. It extracts single GM2 molecules from membranes and presents them in soluble form to β-hexosaminidase A for cleavage of *N*-acetyl-d-galactosamine and conversion to GM3 (by similarity). It also has cholesterol transfer activity.^69^ Although phospholipid and sphingolipid metabolism are important in leishmania,^58^ a connection of GM2A with the pathway has not been shown.
PDHX	The protein PDHX represents a small portion of the pyruvate dehydrogenase (PDH) complex. This complex is located in the mitochondrial matrix and is responsible for the utilization of pyruvate, derived from glucose through glycolysis, in most cells and its conversion to acetyl-CoA and carbon dioxide in the central phase of the Krebs cycle.^70,60^
TFRC	The transferrin receptor C (TFRC), also known as CD71, is an integral membrane glycoprotein, ubiquitously expressed, consisting of two identical subunits linked by disulfide bridges. TFRC uptakes iron by endocytosis of the ligand-occupied transferrin and regulates intracellular iron homeostasis.^86^ Human TFRC has not been related to parasitic infections yet, but its contribution to iron metabolism suggests that - as for transferrin and lactoferrin - it could be employed by the parasite as a source of nutrient intake.

**Figure 8:**
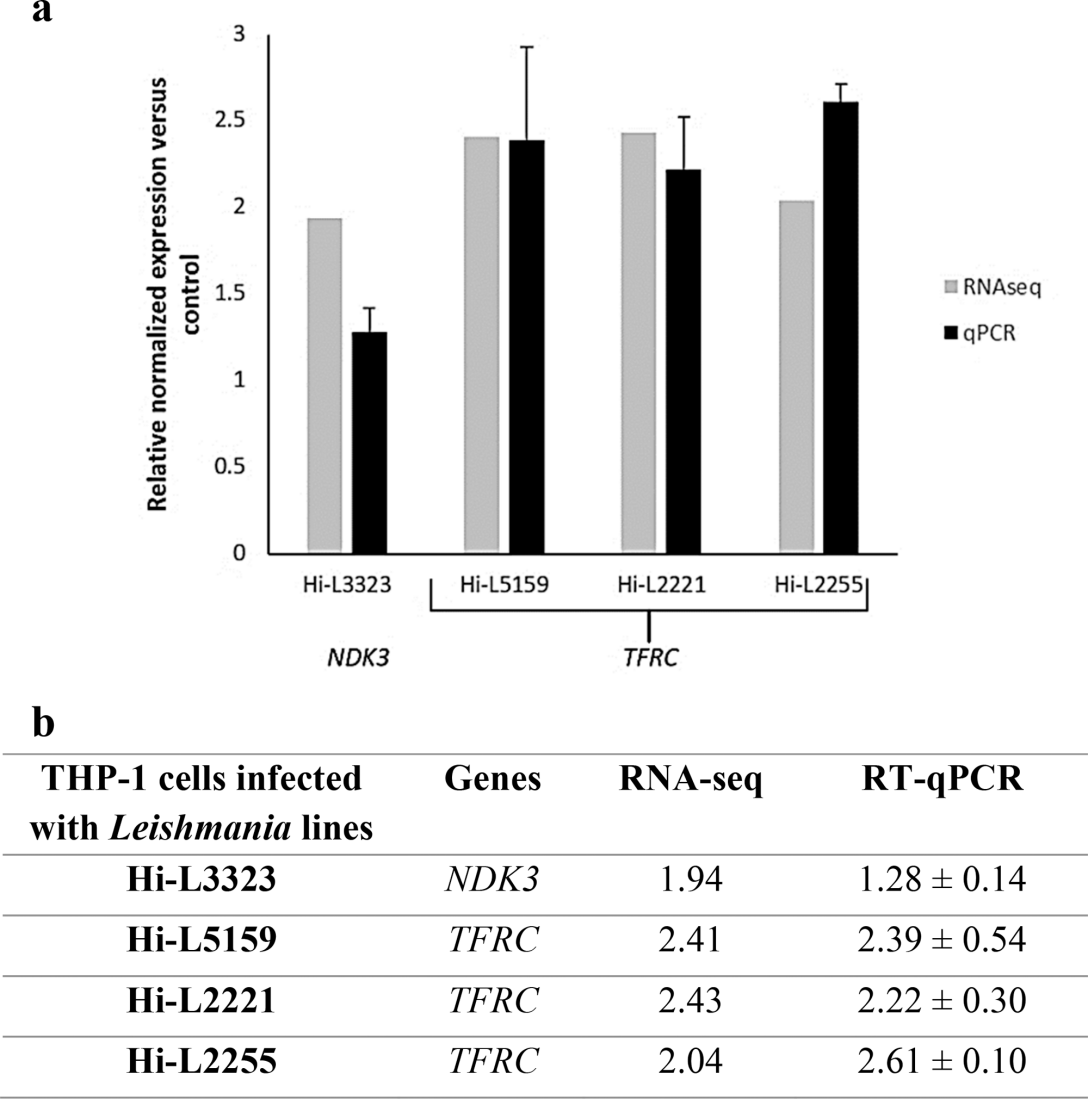
Comparative analysis of the relative expression levels of selected genes determined by RNA-seq and validated by RT-qPCR using a graphical representation (a) and their numerical data (b). The bars (dark gray for RNA-seq and black for RT-qPCR) represent mean ± SD of fold-change expression of *NDK3* and *TFRC* determined from three independent biological replicates analyzed in triplicate. RT-qPCR expression values of the genes in each line were normalized with the expression of ACTB. The relative expression of each macrophage infected with heat-inactivated parasites was set to 1.0.

RT-qPRC was run on the selected samples to validate genes associated with DEPs. Among the six proteins identified, only *TFRC* and *NDK3* were validated. The *TFRC* gene was confirmed to be significantly upregulated in the Hi-L5159 (MIL-resistant) line, and *NDK3* was upregulated in the Hi-L3323 (paromoycin-resistant) line. Furthermore, gene expression experiments revealed the upregulation of *TFRC* in the TF lines Hi-L2221 and Hi-L2255. The results are presented in Figure 8. These findings confirmed that *NDK3* is overexpressed only in drug-resistant cell lines while *TFRC* is present in both drug-resistant (Hi-L5159) and TF (Hi-L2221 and Hi-L2255) cell lines, in contrast to what was found in the proteomic studies. Further studies are needed to confirm these results.

## Conclusions

The proteomic and bioinformatic analyses performed on the infected THP-1 cell lines allowed the characterization of the host metabolic networks that play a significant role in the infected macrophages, with a particular focus on drug resistance mechanisms and TF. The overall research presented in this work has led to the identification of two main proteins that according to our LC-MS/MS proteomic results appear to be upregulated in the samples characterized by drug resistance toward antimony, paromomycin, and MIL. Nucleoside diphosphate kinase 3 (NDK3) has been reported as overexpressed by label-free proteomics (ratio ≥ 1.5, *p* value < 0.05) in an antimony-resistant line (Hi-L3323) as well as in a paromomycin-resistant one (Hi-L2126), and its gene trend has been confirmed by transcriptomics experiments too. On the other hand, the transferrin receptor protein (TFRC) has been identified only in host cells infected with the MIL drug-resistant line by proteomics analysis, whereas gene expression does not suggest the same tendency. An orthogonal transcript expression assay with mRNA sequencing was validated with RT-qPCR for the selected proteins, and upregulated genes encoding for NDK3 and TFRC were validated for the above-mentioned cell lines.

In an optic of a One Health approach, our perspectives include repeating the experiments under the same experimental conditions with a large cohort of clinical isolates of *L. infantum* drug-resistant lines, along with a functional validation with chemical probes which cause downregulation of the two above-mentioned proteins involved in drug resistance to confirm their activity. Despite the degree of complexity surrounding host–guest parasite interactions, proteomics can be a powerful tool to investigate the behavior of complex biological systems. Also, we have demonstrated that by achieving information on over 1300 proteins per cell culture by label-free high-resolution MS, it is possible to isolate biological networks specifically associated with pharmacological response to drugs. In addition, the presented study can be exploited to develop new antileishmaniasis drug targets leveraging host–parasite interactions for the development of new hits or leads through targeted drug discovery programs. The exploitation of the host interactome to model a dual chemotherapeutic strategy has already been proposed to treat bacterial, viral, and parasitic infections.^71−73^ In particular, the *Leishmania*–macrophage cross-interaction network represents an outstanding example of coevolution toward parasite survival and mammalian cell death. By exploiting this peculiar characteristic, a dual guest–host strategy has already been proposed to target hexokinases and histones of drug-resistant *Leishmania* spp. to achieve selective cell death.^73^ Geiger *et al.* and other authors have deepened the focus on the drug discovery exploitation of those biochemical mechanisms involved in the immune escape, with particular attention to metalloproteases, Cruzipain, and sialidases.^74,75^ However, the literature lacks exploitable targets to specificality address drug-resistant lines, and the few host druggable proteins need further validation for crosstalk involvement. In conclusion, with this study we introduce two new upregulated host targets, NDK3 and TFRC, that may be considered for a guest–host strategy designed to overcome drug-resistant strains. If their involvements in drug resistance will be established, the next steps shall include the coadministration of traditional guest-directed molecules with a drug that suppresses NDK3/TFRC host expression to verify the actual synergism of the combination therapy.^76^

## Methods

### Experimental Design

Relying on the assumption that infected host cells could be modulated by the parasites contributing to TF, the purpose of the project is to explore this modulation in THP-1 cells exposed to different *Leishmania* strains from (i) therapeutic failure (TF) *L. infantum* lines from clinical isolates and (ii) *L. infantum* clinical strains characterized by drug resistance to usual antileishmanial chemotherapeutics. In this optic, the analysis was based on the statistical difference (one-way ANOVA) between control groups and each infected cell line. Results were obtained using mass spectrometry, validated with cene expression, and combined with metadata integration and bioinformatic tools.

### Growth of *Leishmania infantum* Lines and THP-1 Cells and *In Vitro* Infection

Human myelomonocytic cells THP-1 were grown at 37 °C and 5% CO_2_ in RPMI-1640 medium supplemented with 10% hiFBS, 2 mM glutamate, 100 units/mL penicillin, and 100 mg/mL streptomycin as described in ref (23). THP-1 cells (30 × 10^6^ cells in 175 cm^2^ flasks) were differentiated to macrophages with 20 ng/mL PMA treatment for 48 h followed by 24 h of culture in fresh medium. All the *L. infantum* lines were grown at 28 °C in RPMI 1640-modified medium (Invitrogen) supplemented with 10% hiFBS (Invitrogen), as described in ref (22). THP-1 cells were infected with the following resistant *L. infantum* lines: *L. infantum* Hi-L3323, an antimony-resistant line isolated from an immunocompromised patient with VL, with a resistance index (RI) versus the Hi-LJPC line in amastigotes higher than 1.6-fold; (ii) *L. infantum* Hi-L2126, a paromomycin-resistant line isolated from another immunocompromised patient with VL, with an RI versus Hi-LJPC in amastigotes higher than 3.9-fold; and (iii) *L. infantum* Hi-L5159, a miltefosine (MIL)-resistant line having an RI versus Hi-LJPC in amastigotes higher than 13.7-fold.^13^ In addition, four strains that were not reported to be drug-resistant but were isolated from TF patients were included in the study: *L. infantum* LLM-2165 and *L. infantum* LLM-2070 isolated from a patient with VL and therapeutic failure; *L. infantum* LLM-2221, resistant to Sb^III^ only in amastigotes; and finally nonresistant *L. infantum* LLM-2255. Control lines were represented by noninfected THP-1 cells and THP-1 cells that were allowed to phagocyte heat-inactivated metacyclic promastigotes from *L. infantum* JPCM5 (MCAN/ES/98/LLM-877) treated at 62 °C for 45 min (Hi-Ldeath). The use of a positive control allowed minimization of the significance of proteins associated with phagolysosome formation and parasitic endocytosis processes not affecting drug-resistance processes.

### Protein Extraction and Sample Processing

Infected THP-1 cells were detached with TrypLE Express reagent. The pellet was then resuspended in 200 μL of lysis buffer (7 M urea, 2 M thiourea, 40 mM Tris, and 4% CHAPS) supplemented with a Complete Mini EDTA-free Protease Inhibitor cocktail 2×. After three freeze–thaw cycles, 150 μL of rehydration buffer (7 M urea, 2 M thiourea, 1% DTT) was added. Then the lysates were homogenized for 30 min and centrifuged at 20000*g* at 4 °C for 45 min to remove debris. The total protein concentration in each lysate supernatant was determined using the Total Protein Kit, Micro Lowry, Peterson’s Modification. THP-1 cells were infected with the above-described *L. infantum* lines, and infectivity profiles were analyzed at 72 h postinfection. For microscopy visualization of intracellular parasites, cells were fixed for 30 min at 4 °C with 2.5% paraformaldehyde in PBS and permeabilized with 0.1% Triton X-100 in PBS for 30 min. We observed ∼56% infection in the *L. infantum* lines, with a median number of 9.7 amastigotes per cell. After sample thawing from −80 °C, the equivalent of 200 μg per line of denatured protein lysates was pipetted onto a filter-aided sample preparation device (FASP) Microcon YM-30 kDa filter (Merck Millipore, Milan, Italy) together with 20 μL of 1 M dithiothreitol (DTT) (Sigma Adrich) and 80 μL of Protease Enhancer (Promega, Milan, Italy). Samples were heat-denatured and then centrifuged at 14000 r.c.f. for 15 min to collect the discharged reagents. Samples were subsequentially treated with 200 μL of 8 M urea/0.1 M Tris-HCl (Sigma-Aldrich) (pH 8.5), centrifuged at 14000 r.c.f. for 15 min, and alkylated with 100 μL of a 55 mM solution of iodoacetamide (IAA) (Sigma-Aldrich) for 20 min at room temperature. After a 14000 r.c.f. centrifugation, filters were drained with two 200 μL cycles of 8 M urea buffer, and the pH was adjusted to 8 with 50 mM ammonium bicarbonate solution (Sigma-Aldrich). Proteins were hydrolyzed overnight with 4 μg of MS sequencing grade trypsin (Promega). Digestion was interrupted with an addition of 5 μL of 1% trifluoroacetic acid (TFA). Tryptic peptides were recovered through 14000*g* spinning for 30 min and a second elution with 40 μL of 0.5 M NaCl. Eluted peptides were diluted with 200 μL of 0.1% aqueous TFA solution and loaded onto Pierce C18 SPE desalting spin columns (Thermo Fisher), previously activated with acetonitrile (ACN) (MS grade, Normapur) and conditioned with the acidic mobile phase. After two wash cycles with 0.1% TFA and 5% aqueous ACN, desalted peptides were eluted with 0.1% formic acid (FA) and 70% ACN and evaporated at room temperature in a SpeedVac (Eppendorf). Dried samples were reconstituted with 60 μL of a mixture of 98% water and 2% acetonitrile with 1% FA, vortexed, and sonicated for 15 min to enhance solubilization.

### LC-MS/MS Analysis

Peptide solutions were transferred in conical vials and analyzed with an UltiMate3000 UHPLC (Thermo Fisher) coupled to an Orbitrap Q-Exactive (Thermo Fisher) high-resolution mass spectrometer (Centro Interdipartimentale Grandi Strumenti, CIGS Unimore). Peptide separation was carried out on a C18-RP Hypersil Gold 100 mm × 2.1 mm column, 1.9 μm particle size (Thermo Fisher Scientific) at a flow rate of 0.5 mL/min. The column oven temperature was set at 30 °C, and the injection volume was 10 μL (25 μg of peptide solution). The mobile phases were (A) 0.1% FA in Milli-Q water and (B) 0.1% FA in MS-grade acetonitrile (Sigma-Aldrich). The following two-step linear gradient was used: 180 min 2% to 28% B followed by 30 min 28% to 40% B. A total run time of 300 min included a final washing step to 98% followed by reconditioning at 2% B. Full-MS/ddMS^2^ (data-dependent fragmentation) was used for mass scanning at *m*/*z* 300–2000 with an isolation window of *m*/*z* 1.5. Each full MS scan was followed by eight data-dependent MS/MS experiments (Top = 8, 28 N for ion fragmentation), and a 6 s dynamic exclusion period was set to maximize the number of fragment ions. Centroided raw data were loaded onto Progenesis QI for Proteomics software (Waters Corporation).^77^ Retention time alignment and intersample normalization were performed on peaks with *m*/*z* between +2 and +6, with a minimum retention time (rt) window of 0.1 min, rt 0.2 to 180 min. Manual 2D vectors were included to adjust technical rt shifts. Data were clustered in replicated batches, and their spectra were exported onto Mascot to perform protein matching through MS/MS ion matching. Detailed information about the LC-MS/MS experiments and methods are provided in Tables S2–S4.

### Preliminary Protein Identification

MS/MS ion search was performed against the SwissProt database (564277 entries, last update October 2021),^42^ the Common Repository of Adventitious Proteins (cRAP) (version 1.0, January 1, 2012),^78^ and an in-house-generated *L. infantum* protein library with a *p* value adjusted to 1% FDR from a reversed-decoy database. Parameters were set as follows: MS tolerance, 10 ppm; MS/MS tolerance, 0.02 Da; fixed modifications, carbamidomethylation (C); variable modifications, oxidation (M) and deamidation (Q, N); missed trypsin cleavages, ≤1. Host proteins with significant sequences in common with the parasite were preventively excluded from the analysis. The *L. infantum* database was obtained from SwissProt (*L. infantum* entry, exported in FASTA format, updated January 2016). Only reviewed proteins were loaded in the final database (51 entries). This search was integrated with manual check of the protein identity. Results were converted into .mzXML extension to perform AUC quantitation on Progenesis QI for Proteomics.^77^ After Mascot results were imported back onto the Progenesis suite, only peptides associated with monocytes’ housekeeping proteins were considered for label-free quantification and intensity normalization. Unique peptides were used for protein abundance calculations on proteins with at least two identified peptides, one of which was unique and nonconflicting. A one-way ANOVA significance test with 5% FDR was performed on each sample against the positive control group. Replicates associated with their *p* value were converted to −log_10_P (“Score”), and ratio values (area of treated/area of control) were converted to fold change values [FC = log_2_(ratio)]. A minimum Score > 1.3 (corresponding to an associated *p* value < 0.05) was used as a significance threshold against an FC ≥ 0.58 (ratio ≥ 1.5) for upregulation or FC ≤ −0.58 (ratio ≤ 1.5^–1^) for downregulation. Specific criteria used in quali-quantitative peptide matching are described in Table S5. The differential analysis between each investigated sample and the control generated a table of 44 significant DEPs, of which 42 were upregulated and two were downregulated with respect to controls. Descriptions of the DEPs are provided in Table S6. Samples were grouped as “drug-resistant” (namely, Hi-L3323, Hi-L2126, and Hi-L5159) and TF (Hi-L2070, Hi-L2165, Hi-L2255, and Hi-L2221), and their DEPs were entered as Universal Protein Knowledgebase (UniProt)^46^ accessions onto the Search Tool for the Retrieval of Interacting Genes/Proteins (STRING)^37^ to generate a protein–protein interaction (PPI) network. A 7-fold enrichment was produced to map the main biological processes involved as Gene Ontology entries (GOs). The network statistics are given in the basic network (Figure 4) and the enriched version with functional GOs (Figure 5) and in Figure S1. Table S9 contains the basic description of the enriched network.

### Network Analysis and Enrichment

The 18 DETs identified and validated by García-Hernández *et al.*(22) and Perea-Martínez *et al.*(23) through transcriptomic experiments were converted into their corresponding proteins through UniProt.^46^ The output was integrated into our interacting STRING database^37^ to measure the level of interconnection between gene regulation and protein expression. A further step on STRING led to the generation of islands within the network based on the general biochemistry pathway description (Figure S1), still without strictly considering the Gene Ontology^38^ (GO) annotations given by the software. GO annotations explain, via the GO terms, the function of a gene at the molecular level (“Molecular Function”), where the gene is active inside the cell (“Cellular Component”), and the pathways to which it contributes (“Biological Process”). These statements are supported by evidence from scientific literature and are currently updated with the newest discoveries. To better visualize the STRING network outcome, the 60 proteins were also input into the Bioinformatics & Evolutionary Genomics toolbar,^54^ which allowed a custom Venn diagram to be calculated and drawn that showed which proteins were overlapping between the two datasets. The tool not only produces a graphical output but also generates a textual output indicating which elements are in the intersection or are unique to one of the two lists.

### Proteomics and Transcriptomics Comparison

We investigated the role of GO “Biological Process” annotations associated with the DEPs, processing them on Microsoft Excel. Transcriptomic work GO annotations for “Biological Process” were given by García-Hernández *et al.*(22) and Perea-Martínez *et al.*,^23^ while our proteomic work GO annotations were searched in the GeneCards database to compare the proteomics and transcriptomics datasets and to find proteins belonging to overlapping biological pathways. Within Microsoft Excel two tables were created (“UNIMORE; GO ID; Qualified GO term” and “NEYRA; GO ID; Qualified GO term”) and were compared using different Excel tools to validate the result. The two GO ID columns had to be compared to find out duplicate values. Two different formulas were used. In the first, COUNTIF was combined with conditional formatting to highlight overlapping GO annotations between the two datasets and allow data to be visualized by colors. In a second moment, the same analysis was run with IF(ISERROR(MATCH) formula, again combined with the former conditional formatting. The two methods returned the same results. Eighteen GO numbers were highlighted in the comparison, but duplicate values were present, so in the Excel “Data” section the “Remove Duplicates” action was performed to obtain a final number of 10 GO annotations found to be shared between the UNIMORE database and the IPBLN database.^38^ Then they were linked to their corresponding proteins.

### GO Biological Process Analysis

The overall 60 proteins were entered into Protein Analysis Through Evolutionary Relationships (PANTHER),^57^ Classification System “Gene List Analysis”. A first “Functional classification viewed in bar chart” analysis was performed, which resulted in a Biological Process bar chart, while a second data manipulation aimed to generate a “Functional classification viewed in gene list”. The list of genes obtained was converted into “Panther Ontology Terms” that include GO “Biological Process” annotations. The “Panther Ontology Terms” list gave results in terms of both GO IDs (PANTHER GO Slim) and PANTHER Protein Class IDs. The latter list was compared with the 10 GO annotations individuated in the former research step. The PC IDs were deleted, and only GO annotations were kept for comparison. Four overlapping pathways were found: iron ion transport (GO:0006826, TFRC); cellular iron ion homeostasis (GO:0006879, TFRC); extracellular matrix organization (GO:0030198, COL4A1 and PECAM1); and transmembrane transport (GO:0055085, TAP1). In brackets are reported respectively the GO annotation and the protein(s) involved in the pathway. The original 60 proteins were entered in the REACTOME “Analysis Tool”.^59,61^ The proteins, submitted as UniProt names, were mapped to pathways, and overrepresentation and pathway topology analyses were run. Overrepresentation analysis is a statistical test that determines whether certain REACTOME pathways are overrepresented (enriched) in the submitted data. The ReacFoam tool on REACTOME allows us to visualize our data with a Voronoi diagram or tessellation, which leads to an interactive overview of the pathway analysis results.^59^

### Biological Significance of Proteins to Validate

A query was set up on the GeneCards,^79^ UniProt,^46^ NCBI,^80^ and OMIM^81^ databases to collect every alias, official name, and accession code recorded for each protein and transcript. These databases were investigated to collect every alias, official name, and accession code recorded for each protein. On the above-mentioned databases a background literature review was conducted to understand the role played by each protein and its location in the cellular organization. Moreover, the publication survey was intended to compare the results of our experiments with the published results. After this process, each protein name was entered in various databases and scientific publications tool bar together with the terms “*Leishmania*” and “leishmaniasis” to explore their involvement in the disease.^79,82^ The results obtained so far have been searched on freely available online tools, namely, PubMed, American Chemical Society (ACS), and others, to find a solid connection between the proteins found and *Leishmania* species infection, an involvement of the proteins in resistance to treatment (antimony, paromomycin, miltefosine) if cell lines are used, and an involvement in therapeutic failure if infected patients are studied.

### Transcriptomic RT-qPRC Analysis

Total RNA (2 μg) was retrotranscribed to cDNA with the qScript cDNA Synthesis Kit (Quanta Biosciences, Inc.) following the manufacturer’s instructions. The specific primer pairs, designed by using the Primer3 software^83^ and used to amplify cDNA, were 5′-ATTGCCGACAGGATGCAGAA-3′ and 5′-GCTGATCCACATCTGCTGGAA-3′ for *ACTB*, 5′-ATGCTGCTTTCCCTTTCCTT-3′ and 5′-CGTGCCACTTTGTTCAACTC-3′ for *TFRC*, and 5′-GGCACTGGCTGTATGAGTAG-3′ and 5′-GTCCAAAGGGATGCTCCAA-3′ for *NDK3*.

Standard curves for each primer pair were generated with 2-fold serial dilutions of the synthesized cDNA to determine primer efficiency. Quantitative PCR was performed in a CFX96 cycler (BioRad). Each 10 μL reaction was set up containing 5 μL of PerfeCta SYBR Green SuperMix (Quanta Biosciences), each primer at 500 nM, and 2 μL of a 1:4 dilution of the synthesized cDNA. All reactions were performed in triplicate, and the specificity of the amplification was verified by melting curve analysis. Gene expression data were normalized to the expression of the reference gene *ACTB* and relative to the control sample using the CFX Manager software with ΔΔ*C*_T_ method.^84,85^

### Data Analysis

Peptide qualitative matching on Mascot MS/MS ion searches was performed with a *p* value of <0.01 adjusted to 1% FDR. The mass tolerance was set to 10 ppm for precursor ions and 0.02 Da for fragment ions. A reversed-sequence decoy database was used to minimize matching error. Each biological sample was analyzed in duplicate, and one-way ANOVA was applied to find DEPs between treated and control groups (FC ≥ 1.5, *p* value < 0.05). The FC for RNaseq was set at ≥2 with a *p* value of <0.05 to determine DETs. qPCR reactions were performed in triplicate, and the specificity of the amplification was verified by melting curve analysis. STRING networks were generated with medium confidence (0.400) and a PPI enrichment *p* value of <0.002. For the biological process comparison in the Excel worksheet, the two groups, proteins and transcripts, were compared using two formulas: =COUNTIF (F2:F79;B2)=1 and IF(ISERROR(MATCHF2;$B$2:$B$535;0));"";F2). When applied, these formulas find duplicate values in the two columns.

## Data Availability

All of the data underlying this study are available in the published article and its Supporting Information. Original MS files containing raw data are available on the public repository Fairdom at the following URL: https://fairdomhub.org/data_files/6280.

## Supporting Information Available

Characteristics of promastigote lines (Table S1); chromatographic parameters employed for peptide separation on the UltiMate 3000 UHPLC (Table S2); UHPLC separation gradient (Table S3); Orbitrap Q Exactive Hybrid MS settings (Table S4); qualitative and quantitative parameters employed for MS peptide matching (Table S5); list of DEPs identified in the ANOVA test from MS proteomics and their corresponding cell lines (Table S6); proteomics fold-change values from one-way ANOVA of DEPs (Table S7); list of DETs identified in the ANOVA test from transcriptomic analysis and their corresponding cell lines (Table S8); description of the proteins from the enriched network referred to Figure 5 (Table S9); enriched STRING network of DEPs and DETs (Figure S1) (PDF)
Excel table of raw MS data (XLSX)
