# Supplementary material for: Label-Free Mass Spectrometry Proteomics Reveals Different Pathways Modulated in THP-1 Cells Infected with Therapeutic Failure and Drug Resistance Leishmania infantum Clinical Isolates
Source: ACS Infect Dis. 2023 Feb 10;9(3):470–85. doi: 10.1021/acsinfecdis.2c00457 (PMC10012269; doi:10.1021/acsinfecdis.2c00457)
Supplement: Supplementary file 1 — id2c00457_si_001.pdf [file id2c00457_si_001.pdf]

SUPPORTING INFORMATION

Label free Mass spectrometry proteomics reveals different pathways modulated in THP-1 cells infected with therapeutic failure and drug resistance *Leishmania infantum* clinical isolates

Lorenzo Tagliazucchi [1,2], Ana Perea-Martinez [3], Greta Fiorini [1], José Ignacio Manzano [3], Filippo Genovese [4], Raquel García-Hernández [3], Diego Pinetti [4], Francisco Gamarro [3], Maria Paola Costi [1 *]

1. Department of Life Science, University of Modena and Reggio Emilia, Via Campi 103, 41125-Modena, Italy;
2. Clinical and Experimental Medicine (CEM) PhD Program, University of Modena and Reggio Emilia, Via Campi 287, 41125-Modena, Italy;
3. Instituto de Parasitología y Biomedicina ''López-Neyra'' (IPBLN-CSIC), Avda. del Conocimiento 17, 18016-Armilla (Granada), Spain;
4. Centro Interdipartimentale Grandi Strumenti (CIGS), University of Modena and Reggio Emilia, Modena, Italy;

* Corresponding authors: pacogamarroc@gmail.com (F.G.); mariapaola.costi@unimore.it (M.P.C.)

Table of content

**Table S1**	Promastigote lines characteristics
**Table S2**	Chromatographic parameters employed for peptide separation on UltiMate 3000 UHPLC (Thermo Fisher).
**Table S3**	UHPLC Separation gradient
**Table S4**	Orbitrap Q Exactive Hybrid (Thermo Fisher) MS settings.
**Table S5**	Qualitative and quantitative parameters employed for MS peptide matching.
**Table S6**	List of the proteins identified as differentially expressed in the ANOVA test from MS proteomics and their corresponding cell lines.
**Table S7**	Proteomics fold-change values from One-way ANOVA (Progenesis) of the differentially expressed Proteins (DEP).
**Table S8**	List of the transcripts identified as differentially expressed in the ANOVA test from transcriptomic analysis (DET) and their corresponding cell lines.
**Table S9**	Description of the proteins from the enriched network referred to Figure 5 from main text.
**Figure S1**	STRING enriched network of differentially expressed proteins and transcripts.

Table S1. *L. infantum* lines employed to infect THP-1 cells and their general characteristics, e.g. resistance levels.

Leishmania strain	Characteristics
**Hi-L3323**	THP-1 infected with L. infantum LEM3323, an antimony-resistant line isolated from an immunodepressed patient with VL (Hospitalier Universitaire of Montpellier and Nimes, France).
**Hi-L2126**	THP-1 infected with L. infantum LEM-2126, a paromomycin-resistant line isolated from an immunodepressed patient with VL (isolate known as Laporta-M, from Hospital General Universitario – Elche, Spain).
**Hi-L5159**	THP-1 infected with L. infantum LEM5159, a miltefosine-resistant line isolated from an immunodepressed patient with VL (Hospitalier Universitaire of Montpellier and Nimes, France).
**Hi-L2165**	THP-1 infected with L. infantum LLM-2165 (clinical isolate known as Checo-S, from Hospital de Fuenlabrada – Madrid; Spain), a line not resistant to drugs, isolated from an immunodepressed patient with VL and therapeutic failure.
**Hi-L2221**	THP-1 infected with L. infantum LLM-2221 (clinical isolate known as Ibe-S, from Hospital de Fuenlabrada – Madrid, Spain), a line not resistant to drugs (in promastigotes), but resistant to miltefosine and antimony in intracellular amastigotes (2-fold Resistant Index to MIL and 3-fold Resistant Index to Antimony, versus their initial isolate) isolated from an immunodepressed patient with VL and therapeutic failure.
**Hi-L2070**	THP-1 infected with L. infantum LLM-2070 (clinical isolate known as Martin-S2, from Hospital de Poniente – Almeria, Spain) a line not resistant to drugs, isolated from an immunodepressed patient with VL and therapeutic failure.
**Hi-L2255**	THP-1 infected with L. infantum LLM-2255 (clinical isolate known as Mialfe-S, from Hospital de Fuenlabrada – Madrid, Spain), a line not resistant to drugs, with higher virulence versus control (in promastigotes, ratio host/parasites 1:2- 3.9-fold more infective; ratio host/parasites 1:5- 2.3-fold more infective; no differences in amastigotes), isolated from an immunodepressed patient with VL and therapeutic failure.
**Ji-Ldeath**	THP-1 infected with Heat-killed metacyclic promastigotes from Leishmania infantum JPCM5 (MCAN/ES/98/LLM-877) (at 62C for 45 min).

Table S2. Chromatographic parameters employed for peptide separation on UltiMate 3000 UHPLC (Thermo Fisher).

**LC PARAMETERS**	
UHPLC	UltiMate3000 (ThermoFisher)
Mass Spectrometer	Orbitrap Q-Exactive (ThermoFisher)
Chromatographic column	C18-RP Hypersil Gold (TF), 100x21mm, 1.9um particle size
Flow Rate	0.5mL/min
Mobile Phases	HCOOH 0.1%aq (A); Acetonitrile (B)
Column temp	30˚C

Table S3. UHPLC Separation gradient

**Gradient**	
t (min)	% B (ACN)
0	2
0.5	2
180	28
210	40
240	98
270	98
300	2

Table S4. Orbitrap Q Exactive Hybrid (Thermo Fisher) MS settings.

MS PARAMETERS	
Default charge:	2+
FullMS	
Microscans	1
Resolution	70000
ACG target	3.00E+06
Max IT	234 s
Scan Range	300 to 2000m/z
Spectrum data	Centroid
ddMS/MS	
Microscans	1
Resolution	17500
ACG target	2.00E+05
Max IT	120 ms
Loop count	8
MSX	1
Top N	8
Is window	1.5 m/z
Is offset	0.4 m/z
(N)CE	28
Spectrum data	Centroid
dd settings	
Min ACG target	1.10E+03
Intensity threshold	9.20E+03
Apex trigger	1 to 24 s
Charge exclusion	1, unassigned
Exclude isotope	on
Dynamic exclusion	6.0s

Table S5. Qualitative and quantitative parameters employed for MS peptide matching both in Mascot Matrix and in Progenesis QI for Proteomics (Nonlinear, Progenesis).

(Mascot)- QUALITATIVE ANALYSIS	
Databases:	SwissProt
	cRAP (common contaminants, ie skin keratins etc)
	Leishmania (S. Ferrari)
Fixed modifications	Carbamidomethylation (C)
Variable modifications	Oxidation (M)
	Deamidation (N,Q)
MS^1 tolerance	10ppm
ms/ms tolerance	0.02 Da
Absolute match tolerance:	p val 0.01 adj to FDR <1%
Enzyme:	Trypsin
Missed cleavages	max_1
# of ^{13}C (isotope tolerance)	1
Decoy database(s)	reversed
m/z range	200 to 2000 m/z
charge(s)	2,3,4 +
QUANTITATIVE ANALYSIS (Progenesis)	
Runs alignment:	automatic
Rt considered peaks	0 to 210 mins
Rt minimum windows	0.1min
Min FC	1.5
Charges	2,3,4
Abundance normalization	Human proteins ms1 peptides
Quantification	Unique (non-conflicting peptides)

Table S6. List of the proteins identified as differentially expressed in the ANOVA test from MS proteomics and the corresponding strain in which they were identified.

UniProt NAME	GENE (UniProt)	PROTEIN ACCESSION (UniProt)	PROTEIN NAME	*L.infantum* Strain
			**Proteins**	
Q13232	**NME3**	NDK3_HUMAN	Nucleoside diphosphate kinase 3 OS=Homo sapiens OX=9606 GN=NME3 PE=1 SV=2	Hi-L3323; Hi-L2126
P15586	**GNS**	GNS_HUMAN	N-acetylglucosamine-6-sulfatase OS=Homo sapiens OX=9606 GN=GNS PE=1 SV=3	Hi-L3323; Hi-L2126; Hi-L2070
P16284	**PECAM1**	PECA1_HUMAN	Platelet endothelial cell adhesion molecule OS=Homo sapiens OX=9606 GN=PECAM1 PE=1 SV=2	Hi-L3323
P13995	**MTHFD2**	MTDC_HUMAN	Bifunctional methylenetetrahydrofolate dehydrogenase/cyclohydrolase, mitochondrial OS=Homo sapiens OX=9606 GN=MTHFD2 PE=1 SV=2	Hi-L2126
P51149	**RAB7A**	RAB7A_HUMAN	Ras-related protein Rab-7a OS=Homo sapiens OX=9606 GN=RAB7A PE=1 SV=1	Hi-L2126
O00560	**SDCBP**	SDCB1_HUMAN	Syntenin-1 OS=Homo sapiens OX=9606 GN=SDCBP PE=1 SV=1	
P08134	**RHOC**	RHOC_HUMAN	Rho-related GTP-binding protein RhoC OS=Homo sapiens OX=9606 GN=RHOC PE=1 SV=1	Hi-L5159
Q96AG4	**LRRC59**	LRC59_HUMAN	Leucine-rich repeat-containing protein 59 OS=Homo sapiens OX=9606 GN=LRRC59 PE=1 SV=1	Hi-L5159
Q86UX7	**FERMT3**	URP2_HUMAN	Fermitin family homolog 3 OS=Homo sapiens OX=9606 GN=FERMT3 PE=1 SV=1	Hi-L5159
O43776	**NARS1**	SYNC_HUMAN	Asparagine--tRNA ligase, cytoplasmic OS=Homo sapiens OX=9606 GN=NARS PE=1 SV=1	Hi-L5159
P62191	**PSMC1**	PRS4_HUMAN	26S proteasome regulatory subunit 4 OS=Homo sapiens OX=9606 GN=PSMC1 PE=1 SV=1	Hi-L5159
P50995	**ANXA11**	ANX11_HUMAN	Annexin A11 OS=Homo sapiens OX=9606 GN=ANXA11 PE=1 SV=1	Hi-L5159; Hi-L2165
P26583	**HMGB2**	HMGB2_HUMAN	High mobility group protein B2 OS=Homo sapiens OX=9606 GN=HMGB2 PE=1 SV=2	Hi-L5159
P36222	**CHI3L1**	CH3L1_HUMAN	Chitinase-3-like protein 1 OS=Homo sapiens OX=9606 GN=CHI3L1 PE=1 SV=2	Hi-L5159; Hi-L2165
Q13627	**DYRK1A**	DYR1A_HUMAN	Dual specificity tyrosine-phosphorylation-regulated kinase 1A OS=Homo sapiens OX=9606 GN=DYRK1A PE=1 SV=2	DR lines, Hi-L2165; Hi-L2221, Hi-L2070
P35606	**COPB2**	COPB2_HUMAN	Coatomer subunit beta' OS=Homo sapiens OX=9606 GN=COPB2 PE=1 SV=2	Hi-L5159
P23219	**PTGS1**	PGH1_HUMAN	Prostaglandin G/H synthase 1 OS=Homo sapiens OX=9606 GN=PTGS1 PE=1 SV=2	Hi-L5159
Q9BT09	**CNPY3**	CNPY3_HUMAN	Protein canopy homolog 3 OS=Homo sapiens OX=9606 GN=CNPY3 PE=1 SV=1	Hi-L5159
Q9NUQ9	**CYRIB**	FA49B_HUMAN	Protein FAM49B OS=Homo sapiens OX=9606 GN=FAM49B PE=1 SV=1	Hi-L5159
P62263	**RPS14**	RS14_HUMAN	40S ribosomal protein S14 OS=Homo sapiens OX=9606 GN=RPS14 PE=1 SV=3	Hi-L5159
P62888	**RPL30**	RL30_HUMAN	60S ribosomal protein L30 OS=Homo sapiens OX=9606 GN=RPL30 PE=1 SV=2	Hi-L5159
Q13740	**ALCAM**	CD166_HUMAN	CD166 antigen OS=Homo sapiens OX=9606 GN=ALCAM PE=1 SV=2	Hi-L5159
P08567	**PLEK**	PLEK_HUMAN	Pleckstrin OS=Homo sapiens OX=9606 GN=PLEK PE=1 SV=3	Hi-L5159
P02786	**TFRC**	TFR1_HUMAN	Transferrin receptor protein 1 OS=Homo sapiens OX=9606 GN=TFRC PE=1 SV=2	Hi-L5159
Q9BSJ8	**ESYT1**	ESYT1_HUMAN	Extended synaptotagmin-1 OS=Homo sapiens OX=9606 GN=ESYT1 PE=1 SV=1	Hi-L5159
P61513	**RPL37A**	RL37A_HUMAN	60S ribosomal protein L37a OS=Homo sapiens OX=9606 GN=RPL37A PE=1 SV=2	Hi-L5159
Q14258	**TRIM25**	TRI25_HUMAN	E3 ubiquitin/ISG15 ligase TRIM25 OS=Homo sapiens OX=9606 GN=TRIM25 PE=1 SV=2	Hi-L5159
P08574	**CYC1**	CY1_HUMAN	Cytochrome c1, heme protein, mitochondrial OS=Homo sapiens OX=9606 GN=CYC1 PE=1 SV=3	Hi-L5159
P53597	**SUCLG1**	SUCA_HUMAN	Succinate--CoA ligase [ADP/GDP-forming] subunit alpha, mitochondrial OS=Homo sapiens OX=9606 GN=SUCLG1 PE=1 SV=4	Hi-L2221
P83111	**LACTB**	LACTB_HUMAN	Serine beta-lactamase-like protein LACTB, mitochondrial OS=Homo sapiens OX=9606 GN=LACTB PE=1 SV=2	Hi-L2165
O95573	**ACSL3**	ACSL3_HUMAN	Long-chain-fatty-acid--CoA ligase 3 OS=Homo sapiens OX=9606 GN=ACSL3 PE=1 SV=3	Hi-L2165; Hi-L2070
P33121	**ACSL1**	ACSL1_HUMAN	Long-chain-fatty-acid--CoA ligase 1 OS=Homo sapiens OX=9606 GN=ACSL1 PE=1 SV=1	Hi-L2070
P17900	**GM2A**	SAP3_HUMAN	Ganglioside GM2 activator OS=Homo sapiens OX=9606 GN=GM2A PE=1 SV=4	Hi-L2165; Hi-L2255
Q7L1Q6	**BZW1**	BZW1_HUMAN	Basic leucine zipper and W2 domain-containing protein 1 OS=Homo sapiens OX=9606 GN=BZW1 PE=1 SV=1	Hi-L2165
Q03518	**TAP1**	TAP1_HUMAN	Antigen peptide transporter 1 OS=Homo sapiens OX=9606 GN=TAP1 PE=1 SV=2	Hi-L2070

Q96C19	**EFHD2**	EFHD2_HUMAN	EF-hand domain-containing protein D2 OS=Homo sapiens OX=9606 GN=EFHD2 PE=1 SV=1	Hi-L2070
P68871	**HBB**	HBB_HUMAN	Hemoglobin subunit beta OS=Homo sapiens OX=9606 GN=HBB PE=1 SV=2	Hi-L2070
P42766	**RPL35**	RL35_HUMAN	60S ribosomal protein L35 OS=Homo sapiens OX=9606 GN=RPL35 PE=1 SV=2	Hi-L2255
Q07020	**RPL18**	RL18_HUMAN	MICOS complex subunit MIC60 OS=Homo sapiens OX=9606 GN=IMMT PE=1 SV=1	Hi-L2255
O00330	**PDHX**	ODPX_HUMAN	Pyruvate dehydrogenase protein X component, mitochondrial OS=Homo sapiens OX=9606 GN=PDHX PE=1 SV=3	Hi-L2255
Q7KZ85	**SUPT6H**	SPT6H_HUMAN	Transcription elongation factor SPT6 OS=Homo sapiens OX=9606 GN=SUPT6H PE=1 SV=2	Hi-L2070

Table S7. Proteomics fold-change values from One-way ANOVA (Progenesis) of the differentially expressed Proteins (DEP), emerged from MS analysis (FC≥ 1.5, and associated p-val< 0.05), used to build the Heat Map. The cross symbol (x) is referred to protein showing FC<1.5 or p-values >0.05 for clarity. All original data are reported in the MS Proteomic Table (Supporting Information 2) containing raw MS files are available on the public repository Fairdom at the URL: https://fairdomhub.org/data_files/6280

UniProt NAME	GENE (UniProt)	Hi-L3323	Hi-L2126	Hi-L5159	HiL-2070	Hi-L2165	Hi-L2221	Hi-L2255
**P49591**	SARS1	0.73	x	x	x	x	.	x
**Q6XQN6**	NAPRT	x	x	x	x	x	x	x
**Q14108**	SCARB2	1.03	x	x	x	x	x	x
**Q13232**	NDK3	1.59	2.48	x	x	x	x	x
**P15586**	GNS	1.18	1.29	x	x	x	1.2	x
**P16284**	PECAM1	0.78	x	x	x	x	x	x
**P13995**	MTHFD2	x	1.23	x	x	x	x	x
**P51149**	RAB7A	x	0.59	x	x	x	x	x
**O00560**	SDCBP	x	1.75	x	x	x	x	x
**P08134**	RHOC	x	x	0.78	x	x	x	x
**Q96AG4**	LRRC59	x	x	1.75	x	x	x	x
**Q86UX7**	FERMT3	x	x	0.98	x	x	x	x
**O43776**	NARS1	x	x	1.39	x	x	x	x
**P62191**	PSMC1	x	x	1.02	x	x	x	x
**P50995**	ANXA11	x	x	0.83	0.68	x	x	x
**P26583**	HMGB2	x	x	1.04	x	x	x	x
**P36222**	CHI3L1	x	x	1.65	1.25	x	x	x
**P35606**	COPB2	x	x	1.92	x	x	x	x
**P23219**	PTGS1	x	x	1.04	x	x	x	x
**Q9BT09**	CNPY3	x	x	1.44	x	x	1.25	x
**Q9NUQ9**	CYRIB_FAM49B	x	x	0.81	x	x	x	x
**P62263**	RPS14	x	x	0.88	x	x	x	x
**P62888**	RPL30	x	x	0.98	x	x	x	x
**Q13740**	ALCAM	x	x	1.62	x	x	x	x
**P08567**	PLEK	x	x	1.34	x	x	x	x
**P02786**	TFRC	x	x	2.69	x	x	x	x
**Q9BSJ8**	ESYT1	x	x	1.23	x	x	x	x
**P61513**	RPL37A	x	x	2.37	x	x	x	x
**Q14258**	TRIM25	x	x	1.36	x	x	x	x
**P08574**	CYC1	x	x	0.81	x	x	x	x
**P53597**	SUCLG1	x	x	x	x	0.59	x	x
**P83111**	LACTB	x	x	x	1.43	x	x	x
**O95573**	ACSL3	x	x	x	0.94	x	x	x
**P33121**	ACSL1	x	x	x	x	x	1.61	x
**P17900**	GM2A	x	x	x	2.05	x	x	2.36
**Q7L1Q6**	BZW1	x	x	x	2.82	x	x	x
**Q03518**	TAP1	x	x	x	x	x	2.37	x
**Q96C19**	EFHD2	x	x	x	x	x	0.64	x
**P68871**	HBB	x	x	x	x	x	2.62	x
**P42766**	RPL35	x	x	x	x	x	x	-0.82

**Q07020**	RPL18	x	x	x	x	x	x	-0.62
**O00330**	PDHX	x	x	x	x	x	x	2.36
**Q7KZ85**	SUPT6H	x	x	x	x	x	3.14	x

Table S8. List of the 18 transcripts identified as differentially expressed in the ANOVA test from transcriptomic analysis in A. Perea-Martínez, 2022, and García-Hernández, 2022, their corresponding encoded proteins used for the STRING Network Analysis (see Figure 4, main text), and their function description [1,2]

Protein name (Uniprot format)	Gene Name	Protein accession entered in STRING Suite	Full name from UniProti
P45844	**ABCG1**	ABCG1_HUMAN	ATP-binding cassette sub-family G member 1
Q9UNQ0	**ABCG2**	ABCG2_HUMAN	Broad substrate specificity ATP-binding cassette transporter ABCG2
Q75V66	**ANO5**	ANO5_HUMAN	NDK3_HUM+C54:D71AN
O43315	**AQP9**	AQP9_HUMAN	Aquaporin-9
Q02338	**BDH1**	BDH_HUMAN	D-beta-hydroxybutyrate dehydrogenase, mitochondrial
P17213	**BPI**	BPI_HUMAN	Bactericidal permeability-increasing protein
O95992	**CH25H**	CH25H_HUMAN	Cholesterol 25-hydroxylase
Q13231	**CHIT1**	CHIT1_HUMAN	Chitotriosidase-1
P02462	**COL4A1**	CO4A1_HUMAN	Collagen alpha-1(IV) chain
P11511	**CYP19A1**	CP19A_HUMAN	Aromatase
Q9UQ10	**DHDH**	DHDH_HUMAN	Trans-1,2-dihydrobenzene-1,2-diol dehydrogenase
P09467	**FBP1**	F16P1_HUMAN	Fructose-1,6-bisphosphatase 1
P09601	**HMOX1**	HMOX1_HUMAN	Heme oxygenase 1
Q16654	**PDK4**	PDK4_HUMAN	Pyruvate dehydrogenase (acetyl-transferring)] kinase isozyme 4, mitochondrial
Q96FL8	**SLC47A1**	S47A1_HUMAN	Multidrug and toxin extrusion protein 1
Q9BXA5	**SUCNR1**	SUCR1_HUMAN	Succinate receptor 1
P02786	**TFRC**	TFR1_HUMAN	Transferrin receptor protein 1 OS=Homo sapiens OX=9606 GN=TFRC PE=1 SV=2
Q13232	**NDK3**	NDK3_HUMAN	Nucleoside diphosphate kinase 3

Table S9. Description of the proteins from the enriched network (Figure 5, main text).

Protein - Node	Biological function
**ABCG1**	ATP-binding cassette sub-family G member 1; Transporter involved in macrophage lipid homeostasis. Is an active component of the macrophage lipid export complex. Could also be involved in intracellular lipid transport processes. The role in cellular lipid homeostasis may not be limited to macrophages; ATP binding cassette subfamily G
**ABCG2**	ATP-binding cassette sub-family G member 2; High-capacity urate exporter functioning in both renal and extrarenal urate excretion. Plays a role in porphyrin homeostasis as it is able to mediates the export of protoporhyrin IX (PPIX) both from mitochondria to cytosol and from cytosol to extracellular space, and cellular export of hemin, and heme. Xenobiotic transporter that may play an important role in the exclusion of xenobiotics from the brain. Appears to play a major role in the multidrug resistance phenotype of several cancer cell lines. Implicated in the efflux of numerous drugs.
**ACSL1**	Long-chain-fatty-acid--CoA ligase 1; Activation of long-chain fatty acids for both synthesis of cellular lipids, and degradation via beta-oxidation. Preferentially uses palmitoleate, oleate and linoleate; Acyl-CoA synthetase family
**ACSL3**	Long-chain-fatty-acid--CoA ligase 3; Acyl-CoA synthetases (ACSL) activates long-chain fatty acids for both synthesis of cellular lipids, and degradation via beta-oxidation. ACSL3 mediates hepatic lipogenesis (By similarity). Preferentially uses myristate, laurate, arachidonate and eicosapentaenoate as substrates (By similarity). Has mainly an anabolic role in energy metabolism. Required for the incorporation of fatty acids into phosphatidylcholine, the major phospholipid located on the surface of VLDL (very low-density lipoproteins); Belongs to the ATP-dependent AMP-binding enzyme family
**ALCAM**	CD166 antigen; Cell adhesion molecule that mediates both heterotypic cell-cell contacts via its interaction with CD6, as well as homotypic cell-cell contacts. Promotes T-cell activation and proliferation via its interactions with CD6. Contributes to the formation and maturation of the immunological synapse via its interactions with CD6. Mediates homotypic interactions with cells that express ALCAM. Required for normal hematopoietic stem cell engraftment in the bone marrow. Mediates attachment of dendritic cells onto endothelial cells via homotypic interaction.
**ANO5**	Anoctamin-5; Does not exhibit calcium-activated chloride channel (CaCC) activity; Anoctamins
**ANXA11**	Annexin A11; Binds specifically to calcyclin in a calcium-dependent manner (By similarity). Required for midbody formation and completion of the terminal phase of cytokinesis; Belongs to the annexin family
**AQP9**	Aquaporin-9; Forms a channel with a broad specificity. Mediates passage of a wide variety of non-charged solutes including carbamides, polyols, purines, and pyrimidines in a phloretin- and mercury-sensitive manner, whereas amino acids, cyclic sugars, Na(+), K(+), Cl(-), and deprotonated monocarboxylates are excluded. Also permeable to urea and glycerol; Belongs to the MIP/aquaporin (TC 1.A.8) family
**ARCN1**	Coatomer subunit delta; Component of the coatomer, a cytosolic protein complex that binds to dilysine motifs and reversibly associates with Golgi non-clathrin-coated vesicles, which further mediate biosynthetic protein transport from the ER, via the Golgi up to the trans Golgi network. The coatomer complex is required for budding from Golgi membranes,and is essential for the retrograde Golgi-to-ER transport of dilysine-tagged proteins. In mammals, the coatomer can only be recruited by membranes associated to ADP-ribosylation factors (ARFs).
**BDH1**	D-beta-hydroxybutyrate dehydrogenase, mitochondrial; Short chain dehydrogenase/reductase superfamily; Belongs to the short-chain dehydrogenases/reductases (SDR) family
**BPI**	Bactericidal permeability-increasing protein; The cytotoxic action of BPI is limited to many species of Gram-negative bacteria; this specificity may be explained by a strong affinity of the very basic N-terminal half for the negatively charged lipopolysaccharides that are unique to the Gram-negative bacterial outer envelope. Has antibacterial activity against the Gram-negative bacterium P.aeruginosa, this activity is inhibited by LPS from P.aeruginosa; Belongs to the BPI/LBP/Plunc superfamily. BPI/LBP family
**BZW1**	Basic leucine zipper and W2 domain-containing protein 1; Enhances histone H4 gene transcription but does not seem to bind DNA directly
**CH25H**	Cholesterol 25-hydroxylase; Catalyzes the formation of 25-hydroxycholesterol from cholesterol, leading to repress cholesterol biosynthetic enzymes. Plays a key role in cell positioning and movement in lymphoid tissues: 25-hydroxycholesterol is an intermediate in biosynthesis of 7-alpha,25-dihydroxycholesterol (7-alpha,25-OHC), an oxysterol that acts as a ligand for the G protein-coupled receptor GPR183/EBI2, a chemotactic receptor for a number of lymphoid cells.
**CHI3L1**	Chitinase-3-like protein 1; Carbohydrate-binding lectin with a preference for chitin. Has no chitinase activity. May play a role in tissue remodeling and in the capacity of cells to respond to and cope with changes in their environment. Plays a role in T-helper cell type 2 (Th2) inflammatory response and IL-13-induced inflammation, regulating allergen sensitization, inflammatory cell apoptosis, dendritic cell accumulation and M2 macrophage differentiation. Facilitates invasion of pathogenic enteric bacteria into colonic mucosa and lymphoid organs.
**CHIT1**	Chitotriosidase-1; Degrades chitin, chitotriose and chitobiose. May participate in the defense against nematodes and other pathogens. Isoform 3 has no enzymatic activity; Belongs to the glycosyl hydrolase 18 family. Chitinase class II subfamily
**CNPY3**	Protein canopy homolog 3; Toll-like receptor (TLR)-specific co-chaperone for HSP90B1. Required for proper TLR folding, except that of TLR3, and hence controls TLR exit from the endoplasmic reticulum. Consequently, required for both innate and adaptive immune responses (By similarity); Belongs to the canopy family
**COL4A1**	Collagen alpha-1(IV) chain; Type IV collagen is the major structural component of glomerular basement membranes (GBM), forming a 'chicken-wire' meshwork together with laminins, proteoglycans and entactin/nidogen
**COPB1**	Coatomer subunit beta; The coatomer is a cytosolic protein complex that binds to dilysine motifs and reversibly associates with Golgi non- clathrin-coated vesicles, which further mediate biosynthetic protein transport from the ER, via the Golgi up to the trans Golgi network. Coatomer complex is required for budding from Golgi membranes, and is essential for the retrograde Golgi-to-ER transport of dilysine-tagged proteins. In mammals, the coatomer can only be recruited by membranes associated to ADP-ribosylation factors (ARFs), which are small GTP-binding proteins.
**COPB2**	Coatomer subunit beta; The coatomer is a cytosolic protein complex that binds to dilysine motifs and reversibly associates with Golgi non- clathrin-coated vesicles, which further mediate biosynthetic protein transport from the ER, via the Golgi up to the trans Golgi network. Coatomer complex is required for budding from Golgi membranes, and is essential for the retrograde Golgi-to-ER transport of dilysine-tagged proteins. In mammals, the coatomer can only be recruited by membranes associated to ADP-ribosylation factors (ARFs), which are small GTP-binding proteins.
**CYC1**	Cytochrome c1, heme protein, mitochondrial; This is the heme-containing component of the cytochrome b-c1 complex, which accepts electrons from Rieske protein and transfers electrons to cytochrome c in the mitochondrial respiratory chain.
**CYP19A1**	Aromatase; Catalyzes the formation of aromatic C18 estrogens from C19 androgens; Cytochrome P450 family 19
**DHDH**	Dihydrodiol dehydrogenase / d-xylose 1-dehydrogenase (nadp); Dihydrodiol dehydrogenase; Belongs to the Gfo/Idh/MocA family
**DLAT**	Pyruvate dehydrogenase e2 component (dihydrolipoamide acetyltransferase); Dihydrolipoamide S-acetyltransferase; The pyruvate dehydrogenase complex catalyzes the overall conversion of pyruvate to acetyl-CoA and CO(2), and thereby links the glycolytic pathway to the tricarboxylic cycle
**DLD**	Dihydrolipoyl dehydrogenase, mitochondrial; Lipoamide dehydrogenase is a component of the glycine cleavage system as well as an E3 component of three alpha-ketoacid dehydrogenase complexes (pyruvate-, alpha-ketoglutarate-, and branched-chain amino acid-dehydrogenase complex). In monomeric form has additional moonlighting function as serine protease. Involved in the hyperactivation of spermatazoa during capacitation and in the spermatazoal acrosome reaction (By similarity)
**DYRK1A**	Dual specificity tyrosine-phosphorylation-regulated kinase 1A; Dual-specificity kinase which possesses both serine/threonine and tyrosine kinase activities. May play a role in a signaling pathway regulating nuclear functions of cell proliferation. Modulates alternative splicing by phosphorylating the splice factor SRSF6 (By similarity). Exhibits a substrate preference for proline at position P+1 and arginine at position P- 3. Has pro-survival function and negatively regulates the apoptotic process. Promotes cell survival upon genotoxic stress through phosphorylation of SIRT1.
**EEF2**	Elongation factor 2; Catalyzes the GTP-dependent ribosomal translocation step during translation elongation. During this step, the ribosome changes from the pre-translocational (PRE) to the post- translocational (POST) state as the newly formed A-site-bound peptidyl-tRNA and P-site-bound deacylated tRNA move to the P and E sites, respectively. Catalyzes the coordinated movement of the two tRNA molecules, the mRNA and conformational changes in the ribosome; Belongs to the TRAFAC class translation factor GTPase superfamily. Classic translation factor GTPase family. EF-G/EF-2 subfamily

**EFHD2**	EF-hand domain-containing protein D2; May regulate B-cell receptor (BCR)-induced immature and primary B-cell apoptosis. Plays a role as negative regulator of the canonical NF-kappa-B-activating branch. Controls spontaneous apoptosis through the regulation of BCL2L1 abundance; EF-hand domain containing
**ESYT1**	Extended synaptotagmin-1; Binds glycerophospholipids in a barrel-like domain and may play a role in cellular lipid transport (By similarity). Binds calcium (via the C2 domains) and translocates to sites of contact between the endoplasmic reticulum and the cell membrane in response to increased cytosolic calcium levels. Helps tether the endoplasmic reticulum to the cell membrane and promotes the formation of appositions between the endoplasmic reticulum and the cell membrane.
**FAM49B**	Protein FAM49B; Family with sequence similarity 49 member B
**FBP1**	Fructose-1,6-bisphosphatase 1; Catalyzes the hydrolysis of fructose 1,6-bisphosphate to fructose 6-phosphate in the presence of divalent cations, acting as a rate-limiting enzyme in gluconeogenesis. Plays a role in regulating glucose sensing and insulin secretion of pancreatic beta-cells. Appears to modulate glycerol gluconeogenesis in liver.
**FERMT3**	Fermitin family homolog 3; Plays a central role in cell adhesion in hematopoietic cells. Acts by activating the integrin beta-1-3 (ITGB1, ITGB2 and ITGB3) (By similarity). Required for integrin-mediated platelet adhesion and leukocyte adhesion to endothelial cells. Required for activation of integrin beta-2 (ITGB2) in polymorphonuclear granulocytes (PMNs) (By similarity); Belongs to the kindlin family
**GM2A**	Ganglioside GM2 activator; The large binding pocket can accommodate several single chain phospholipids and fatty acids, GM2A also exhibits some calcium-independent phospholipase activity (By similarity). Binds gangliosides and stimulates ganglioside GM2 degradation. It stimulates only the breakdown of ganglioside GM2 and glycolipid GA2 by beta-hexosaminidase A. It extracts single GM2 molecules from membranes and presents them in soluble form to beta- hexosaminidase A for cleavage of N-acetyl-D-galactosamine and conversion to GM3
**GNS**	N-acetylglucosamine-6-sulfatase; Glucosamine-6-sulfatase; Belongs to the sulfatase family
**HBB**	Hemoglobin subunit beta; Involved in oxygen transport from the lung to the various peripheral tissues; Belongs to the globin family
**HMGB2**	High mobility group protein B2; Multifunctional protein with various roles in different cellular compartments. May act in a redox sensitive manner. In the nucleus is an abundant chromatin-associated non-histone protein involved in transcription, chromatin remodeling and V(D)J recombination and probably other processes. Binds DNA with a preference to non-canonical DNA structures such as single-stranded DNA.
**HMOX1**	Heme oxygenase 1; Heme oxygenase cleaves the heme ring at the alpha methene bridge to form biliverdin. Biliverdin is subsequently converted to bilirubin by biliverdin reductase. Under physiological conditions, the activity of heme oxygenase is highest in the spleen, where senescent erythrocytes are sequestrated and destroyed. Exhibits cytoprotective effects since excess of free heme sensitizes cells to undergo apoptosis
**LACTB**	Serine beta-lactamase-like protein LACTB, mitochondrial; Mitochondrial serine protease that acts as a regulator of mitochondrial lipid metabolism. Acts by decreasing protein levels of PISD, a mitochondrial enzyme that converts phosphatidylserine (PtdSer) to phosphatidylethanolamine (PtdEtn), thereby affecting mitochondrial lipid metabolism. It is unclear whether it acts directly by mediating proteolysis of PISD or by mediating proteolysis of another lipid metabolism protein. Acts as a tumor suppressor that has the ability to inhibit proliferation of multiple types of breast cancer cell.
**LRRC59**	Leucine-rich repeat-containing protein 59; Required for nuclear import of FGF1, but not that of FGF2. Might regulate nuclear import of exogenous FGF1 by facilitating interaction with the nuclear import machinery and by transporting cytosolic FGF1 to, and possibly through, the nuclear pores
**MTHFD2**	Bifunctional methylenetetrahydrofolate dehydrogenase/cyclohydrolase, mitochondrial; Although its dehydrogenase activity is NAD-specific, it can also utilize NADP at a reduced efficiency; Belongs to the tetrahydrofolate dehydrogenase/cyclohydrolase family
**NARS**	Asparagine--tRNA ligase, cytoplasmic; Aminoacyl tRNA synthetases, Class II
**NDUFS7**	NADH dehydrogenase [ubiquinone] iron-sulfur protein 7, mitochondrial; Core subunit of the mitochondrial membrane respiratory chain NADH dehydrogenase (Complex I) that is believed to belong to the minimal assembly required for catalysis. Complex I functions in the transfer of electrons from NADH to the respiratory chain.
**NME3**	Nucleoside diphosphate kinase 3; Major role in the synthesis of nucleoside triphosphates other than ATP. The ATP gamma phosphate is transferred to the NDP beta phosphate via a ping-pong mechanism, using a phosphorylated active-site intermediate. Probably has a role in normal hematopoiesis by inhibition of granulocyte differentiation and induction of apoptosis; NME/NM23 family
**PDHB**	Pyruvate dehydrogenase E1 component subunit beta, mitochondrial; The pyruvate dehydrogenase complex catalyzes the overall conversion of pyruvate to acetyl-CoA and CO(2), and thereby links the glycolytic pathway to the tricarboxylic cycle
**PDHX**	Pyruvate dehydrogenase protein X component, mitochondrial; Required for anchoring dihydrolipoamide dehydrogenase (E3) to the dihydrolipoamide transacetylase (E2) core of the pyruvate dehydrogenase complexes of eukaryotes. This specific binding is essential for a functional PDH complex
**PDK4**	[Pyruvate dehydrogenase (acetyl-transferring)] kinase isozyme 4, mitochondrial; Kinase that plays a key role in regulation of glucose and fatty acid metabolism and homeostasis via phosphorylation of the pyruvate dehydrogenase subunits PDHA1 and PDHA2. This inhibits pyruvate dehydrogenase activity, and thereby regulates metabolite flux through the tricarboxylic acid cycle, down-regulates aerobic respiration and inhibits the formation of acetyl-coenzyme A from pyruvate.
**PECAM1**	Platelet endothelial cell adhesion molecule; Cell adhesion molecule which is required for leukocyte transendothelial migration (TEM) under most inflammatory conditions. Tyr-690 plays a critical role in TEM and is required for efficient trafficking of PECAM1 to and from the lateral border recycling compartment (LBRC) and is also essential for the LBRC membrane to be targeted around migrating leukocytes. Heterophilic interaction with CD177 plays a role in transendothelial migration of neutrophils.
**PLEK**	Pleckstrin; Major protein kinase C substrate of platelets; Pleckstrin homology domain containing.
**PSMA2**	Proteasome subunit alpha type-2; Component of the 20S core proteasome complex involved in the proteolytic degradation of most intracellular proteins. This complex plays numerous essential roles within the cell by associating with different regulatory particles. Associated with two 19S regulatory particles, forms the 26S proteasome and thus participates in the ATP-dependent degradation of ubiquitinated proteins. The 26S proteasome plays a key role in the maintenance of protein homeostasis by removing misfolded or damaged proteins that could impair cellular functions.
**PSMA3**	Proteasome subunit alpha type-3; Component of the 20S core proteasome complex involved in the proteolytic degradation of most intracellular proteins. This complex plays numerous essential roles within the cell by associating with different regulatory particles. Associated with two 19S regulatory particles, forms the 26S proteasome and thus participates in the ATP-dependent degradation of ubiquitinated proteins. The 26S proteasome plays a key role in the maintenance of protein homeostasis by removing misfolded or damaged proteins that could impair cellular functions.
**PSMA4**	Proteasome subunit alpha type-4; Component of the 20S core proteasome complex involved in the proteolytic degradation of most intracellular proteins. This complex plays numerous essential roles within the cell by associating with different regulatory particles. Associated with two 19S regulatory particles, forms the 26S proteasome and thus participates in the ATP-dependent degradation of ubiquitinated proteins. The 26S proteasome plays a key role in the maintenance of protein homeostasis by removing misfolded or damaged proteins that could impair cellular functions.
**PSMA5**	Proteasome subunit alpha type-5; Component of the 20S core proteasome complex involved in the proteolytic degradation of most intracellular proteins. This complex plays numerous essential roles within the cell by associating with different regulatory particles. Associated with two 19S regulatory particles, forms the 26S proteasome and thus participates in the ATP-dependent degradation of ubiquitinated proteins. The 26S proteasome plays a key role in the maintenance of protein homeostasis by removing misfolded or damaged proteins that could impair cellular functions.
**PSMA6**	Proteasome subunit alpha type-6; Component of the 20S core proteasome complex involved in the proteolytic degradation of most intracellular proteins. This complex plays numerous essential roles within the cell by associating with different regulatory particles. Associated with two 19S regulatory particles, forms the 26S proteasome and thus participates in the ATP-dependent degradation of ubiquitinated proteins. The 26S proteasome plays a key role in the maintenance of protein homeostasis by removing misfolded or damaged proteins that could impair cellular functions.
**PSMB1**	Proteasome subunit beta type-1; Component of the 20S core proteasome complex involved in the proteolytic degradation of most intracellular proteins. This complex plays numerous essential roles within the cell by associating with different regulatory particles. Associated with two 19S regulatory particles, forms the 26S proteasome and thus participates in the ATP-dependent degradation of ubiquitinated proteins. The 26S proteasome plays a key role in the maintenance of protein homeostasis by removing misfolded or damaged proteins that could impair cellular functions.
**PSMB4**	Proteasome subunit beta type-4; Component of the 20S core proteasome complex involved in the proteolytic degradation of most intracellular proteins. This complex plays numerous essential roles within the cell by associating with different regulatory particles. Associated with two 19S regulatory particles, forms the 26S proteasome and thus participates in the ATP-dependent degradation of ubiquitinated proteins. The 26S proteasome plays a key role in the maintenance of protein homeostasis by removing misfolded or damaged proteins that could impair cellular functions.

**PSMB6**	Proteasome subunit beta type-6; Component of the 20S core proteasome complex involved in the proteolytic degradation of most intracellular proteins. This complex plays numerous essential roles within the cell by associating with different regulatory particles. Associated with two 19S regulatory particles, forms the 26S proteasome and thus participates in the ATP-dependent degradation of ubiquitinated proteins. The 26S proteasome plays a key role in the maintenance of protein homeostasis by removing misfolded or damaged proteins that could impair cellular functions.
**PSMB7**	Proteasome subunit beta type-7; Component of the 20S core proteasome complex involved in the proteolytic degradation of most intracellular proteins. This complex plays numerous essential roles within the cell by associating with different regulatory particles. Associated with two 19S regulatory particles, forms the 26S proteasome and thus participates in the ATP-dependent degradation of ubiquitinated proteins. The 26S proteasome plays a key role in the maintenance of protein homeostasis by removing misfolded or damaged proteins that could impair cellular functions.
**PSMC1**	26S proteasome regulatory subunit 4; Component of the 26S proteasome, a multiprotein complex involved in the ATP-dependent degradation of ubiquitinated proteins. This complex plays a key role in the maintenance of protein homeostasis by removing misfolded or damaged proteins, which could impair cellular functions, and by removing proteins whose functions are no longer required. Therefore, the proteasome participates in numerous cellular processes, including cell cycle progression, apoptosis, or DNA damage repair.
**PSMC4**	26S proteasome regulatory subunit 6B; Component of the 26S proteasome, a multiprotein complex involved in the ATP-dependent degradation of ubiquitinated proteins. This complex plays a key role in the maintenance of protein homeostasis by removing misfolded or damaged proteins, which could impair cellular functions, and by removing proteins whose functions are no longer required. Therefore, the proteasome participates in numerous cellular processes, including cell cycle progression, apoptosis, or DNA damage repair.
**PSMD1**	26S proteasome non-ATPase regulatory subunit 1; Component of the 26S proteasome, a multiprotein complex involved in the ATP-dependent degradation of ubiquitinated proteins. This complex plays a key role in the maintenance of protein homeostasis by removing misfolded or damaged proteins, which could impair cellular functions, and by removing proteins whose functions are no longer required. Therefore, the proteasome participates in numerous cellular processes, including cell cycle progression, apoptosis, or DNA damage repair; Belongs to the proteasome subunit S1 family
**PSMD11**	26S proteasome non-ATPase regulatory subunit 11; Component of the 26S proteasome, a multiprotein complex involved in the ATP-dependent degradation of ubiquitinated proteins. This complex plays a key role in the maintenance of protein homeostasis by removing misfolded or damaged proteins, which could impair cellular functions, and by removing proteins whose functions are no longer required. Therefore, the proteasome participates in numerous cellular processes, including cell cycle progression, apoptosis, or DNA damage repair. In the complex, PSMD11 is required for proteasome assembly.
**PSMD2**	26S proteasome non-ATPase regulatory subunit 2; Component of the 26S proteasome, a multiprotein complex involved in the ATP-dependent degradation of ubiquitinated proteins. This complex plays a key role in the maintenance of protein homeostasis by removing misfolded or damaged proteins, which could impair cellular functions, and by removing proteins whose functions are no longer required. Therefore, the proteasome participates in numerous cellular processes, including cell cycle progression, apoptosis, or DNA damage repair; Belongs to the proteasome subunit S2 family
**PSMD3**	26S proteasome non-ATPase regulatory subunit 3; Component of the 26S proteasome, a multiprotein complex involved in the ATP-dependent degradation of ubiquitinated proteins. This complex plays a key role in the maintenance of protein homeostasis by removing misfolded or damaged proteins, which could impair cellular functions, and by removing proteins whose functions are no longer required. Therefore, the proteasome participates in numerous cellular processes, including cell cycle progression, apoptosis, or DNA damage repair; Belongs to the proteasome subunit S3 family
**PSMD7**	26S proteasome non-ATPase regulatory subunit 7; Component of the 26S proteasome, a multiprotein complex involved in the ATP-dependent degradation of ubiquitinated proteins. This complex plays a key role in the maintenance of protein homeostasis by removing misfolded or damaged proteins, which could impair cellular functions, and by removing proteins whose functions are no longer required. Therefore, the proteasome participates in numerous cellular processes, including cell cycle progression, apoptosis, or DNA damage repair; Belongs to the peptidase M67A family
**PSMD8**	26S proteasome non-ATPase regulatory subunit 8; Component of the 26S proteasome, a multiprotein complex involved in the ATP-dependent degradation of ubiquitinated proteins. This complex plays a key role in the maintenance of protein homeostasis by removing misfolded or damaged proteins, which could impair cellular functions, and by removing proteins whose functions are no longer required. Therefore, the proteasome participates in numerous cellular processes, including cell cycle progression, apoptosis, or DNA damage repair.
**PTGS1**	Prostaglandin G/H synthase 1; Converts arachidonate to prostaglandin H2 (PGH2), a committed step in prostanoid synthesis. Involved in the constitutive production of prostanoids in particular in the stomach and platelets. In gastric epithelial cells, it is a key step in the generation of prostaglandins, such as prostaglandin E2 (PGE2), which plays an important role in cytoprotection. In platelets, it is involved in the generation of thromboxane A2 (TXA2), which promotes platelet activation and aggregation, vasoconstriction and proliferation of vascular smooth muscle cells.
**RAB7A**	Ras-related protein Rab-7a; Key regulator in endo-lysosomal trafficking. Governs early-to-late endosomal maturation, microtubule minus-end as well as plus-end directed endosomal migration and positioning, and endosome-lysosome transport through different protein-protein interaction cascades. Plays a central role, not only in endosomal traffic, but also in many other cellular and physiological events, such as growth-factor-mediated cell signaling, nutrient- transportor mediated nutrient uptake, neurotrophin transport in the axons of neurons and lipid metabolism.
**RHOC**	Rho-related GTP-binding protein RhoC; Regulates a signal transduction pathway linking plasma membrane receptors to the assembly of focal adhesions and actin stress fibers. Serves as a microtubule-dependent signal that is required for the myosin contractile ring formation during cell cycle cytokinesis. Regulates apical junction formation in bronchial epithelial cells; Rho family GTPases
**RPL13**	Large subunit ribosomal protein l13e; Ribosomal protein L13
**RPL15**	Large subunit ribosomal protein l15e; Ribosomal protein L15
**RPL18**	60S ribosomal protein L18; Component of the large ribosomal subunit; Belongs to the eukaryotic ribosomal protein eL18 family
**RPL18A**	Large subunit ribosomal protein l18ae; Belongs to the eukaryotic ribosomal protein eL20 family
**RPL19**	Large subunit ribosomal protein l19e; Ribosomal protein L19; Belongs to the eukaryotic ribosomal protein eL19 family
**RPL30**	Large subunit ribosomal protein l30e; Ribosomal protein L30; Belongs to the eukaryotic ribosomal protein eL30 family
**RPL35**	60S ribosomal protein L35; Component of the large ribosomal subunit
**RPL37**	60S ribosomal protein L37; Binds to the 23S rRNA; L ribosomal proteins
**RPL37A**	Large subunit ribosomal protein l37ae; Ribosomal protein L37a
**RPL38**	Large subunit ribosomal protein l38e; Ribosomal protein L38; Belongs to the eukaryotic ribosomal protein eL38 family
**RPL8**	60S ribosomal protein L8; Component of the large ribosomal subunit
**RPS11**	Small subunit ribosomal protein s11e; Ribosomal protein S11; Belongs to the universal ribosomal protein uS17 family
**RPS12**	Small subunit ribosomal protein s12e; Ribosomal protein S12
**RPS14**	Small subunit ribosomal protein s14e; Ribosomal protein S14
**RPS16**	Small subunit ribosomal protein s16e; Ribosomal protein S16
**RPS23**	40S ribosomal protein S23; Component of the ribosome, a large ribonucleoprotein complex responsible for the synthesis of proteins in the cell. The small ribosomal subunit (SSU) binds messenger RNAs (mRNAs) and translates the encoded message by selecting cognate aminoacyl-transfer RNA (tRNA) molecules. The large subunit (LSU) contains the ribosomal catalytic site termed the peptidyl transferase center (PTC), which catalyzes the formation of peptide bonds, thereby polymerizing the amino acids delivered by tRNAs into a polypeptide chain.

**RPS27A**	Ubiquitin-40S ribosomal protein S27a; Ubiquitin: Exists either covalently attached to another protein, or free (unanchored). When covalently bound, it is conjugated to target proteins via an isopeptide bond either as a monomer (monoubiquitin), a polymer linked via different Lys residues of the ubiquitin (polyubiquitin chains) or a linear polymer linked via the initiator Met of the ubiquitin (linear polyubiquitin chains). Polyubiquitin chains, when attached to a target protein, have different functions depending on the Lys residue of the ubiquitin that is linked.
**RPS3**	40S ribosomal protein S3; Involved in translation as a component of the 40S small ribosomal subunit. Has endonuclease activity and plays a role in repair of damaged DNA. Cleaves phosphodiester bonds of DNAs containing altered bases with broad specificity and cleaves supercoiled DNA more efficiently than relaxed DNA. Displays high binding affinity for 7,8-dihydro-8-oxoguanine (8-oxoG), a common DNA lesion caused by reactive oxygen species (ROS). Has also been shown to bind with similar affinity to intact and damaged DNA.
**RPS9**	Small subunit ribosomal protein s9e; Ribosomal protein S9
**SDCBP**	Syntenin-1; Multifunctional adapter protein involved in diverse array of functions including trafficking of transmembrane proteins, neuro and immunomodulation, exosome biogenesis, and tumorigenesis. Positively regulates TGFB1- mediated SMAD2/3 activation and TGFB1-induced epithelial-to- mesenchymal transition (EMT) and cell migration in various cell types. May increase TGFB1 signaling by enhancing cell-surface expression of TGFR1 by preventing the interaction between TGFR1 and CAV1 and subsequent CAV1-dependent internalization and degradation of TGFR1.
**SLC47A1**	Multidrug resistance protein, mate family; Multidrug and toxin extrusion protein 1; Solute transporter for tetraethylammonium (TEA), 1- methyl-4-phenylpyridinium (MPP), cimetidine, N-methylnicotinamide (NMN), metformin, creatinine, guanidine, procainamide, topotecan, estrone sulfate, acyclovir, ganciclovir and also the zwitterionic cephalosporin, cephalexin and cephradin. Seems to also play a role in the uptake of oxaliplatin (a new platinum anticancer agent). Able to transport paraquat (PQ or N,N-dimethyl-4-4'-bipiridinium); a widely used herbicid.
**SUCLG1**	Succinate--CoA ligase [ADP/GDP-forming] subunit alpha, mitochondrial; Succinyl-CoA synthetase functions in the citric acid cycle (TCA), coupling the hydrolysis of succinyl-CoA to the synthesis of either ATP or GTP and thus represents the only step of substrate-level phosphorylation in the TCA. The alpha subunit of the enzyme binds the substrates coenzyme A and phosphate, while succinate binding and specificity for either ATP or GTP is provided by different beta subunits
**SUCNR1**	Succinate receptor 1; Receptor for succinate
**SUPT6H**	Transcription elongation factor SPT6; Transcription elongation factor which binds histone H3 and plays a key role in the regulation of transcription elongation and mRNA processing. Enhances the transcription elongation by RNA polymerase II (RNAPII) and is also required for the efficient activation of transcriptional elongation by the HIV-1 nuclear transcriptional activator, Tat. Besides chaperoning histones in transcription, acts to transport and splice mRNA by forming a complex with IWS1 and the C-terminal domain (CTD) of the RNAPII subunit RPB1 (POLR2A).
**TAP1**	Antigen peptide transporter 1; Involved in the transport of antigens from the cytoplasm to the endoplasmic reticulum for association with MHC class I molecules. Also acts as a molecular scaffold for the final stage of MHC class I folding, namely the binding of peptide. Nascent MHC class I molecules associate with TAP via tapasin. Inhibited by the covalent attachment of herpes simplex virus ICP47 protein, which blocks the peptide-binding site of TAP. Inhibited by human cytomegalovirus US6 glycoprotein, which binds to the lumenal side of the TAP complex and inhibits peptide translocation.
**TFRC**	Transferrin receptor protein 1; Cellular uptake of iron occurs via receptor-mediated endocytosis of ligand-occupied transferrin receptor into specialized endosomes. Endosomal acidification leads to iron release. The apotransferrin-receptor complex is then recycled to the cell surface with a return to neutral pH and the concomitant loss of affinity of apotransferrin for its receptor. Transferrin receptor is necessary for development of erythrocytes and the nervous system (By similarity).
**TRIM25**	E3 ubiquitin/ISG15 ligase TRIM25; Functions as a ubiquitin E3 ligase and as an ISG15 E3 ligase. Involved in innate immune defense against viruses by mediating ubiquitination of DDX58. Mediates 'Lys-63'-linked polyubiquitination of the DDX58 N-terminal CARD-like region which is crucial for triggering the cytosolic signal transduction that leads to the production of interferons in response to viral infection. Promotes ISGylation of 14-3-3 sigma (SFN), an adapter protein implicated in the regulation of a large spectrum signaling pathway.
**UQCRC1**	Cytochrome b-c1 complex subunit 1, mitochondrial; This is a component of the ubiquinol-cytochrome c reductase complex (complex III or cytochrome b-c1 complex), which is part of the mitochondrial respiratory chain. This protein may mediate formation of the complex between cytochromes c and c1; M16 metallopeptidases
**UQCRC2**	Cytochrome b-c1 complex subunit 2, mitochondrial; This is a component of the ubiquinol-cytochrome c reductase complex (complex III or cytochrome b-c1 complex), which is part of the mitochondrial respiratory chain. The core protein 2 is required for the assembly of the complex; M16 metallopeptidases
**UQCRFS1**	Ubiquinol-cytochrome c reductase, rieske iron-sulfur polypeptide 1; Cytochrome b-c1 complex subunit Rieske, mitochondrial; Cytochrome b-c1 complex subunit Rieske, mitochondrial: Component of the mitochondrial ubiquinol-cytochrome c reductase complex dimer (complex III dimer), which is a respiratory chain that generates an electrochemical potential coupled to ATP synthesis. Incorporation of UQCRFS1 is the penultimate step in complex III assembly (By similarity)
**UQCRH**	Cytochrome b-c1 complex subunit 6, mitochondrial; This is a component of the ubiquinol-cytochrome c reductase complex (complex III or cytochrome b-c1 complex), which is part of the mitochondrial respiratory chain. This protein may mediate formation of the complex between cytochromes c and c1; Belongs to the UQCRH/QCR6 family
**USP14**	Ubiquitin carboxyl-terminal hydrolase 14; Proteasome-associated deubiquitinase which releases ubiquitin from the proteasome targeted ubiquitinated proteins. Ensures the regeneration of ubiquitin at the proteasome. Is a reversibly associated subunit of the proteasome and a large fraction of proteasome-free protein exists within the cell. Required for the degradation of the chemokine receptor CXCR4 which is critical for CXCL12-induced cell chemotaxis.

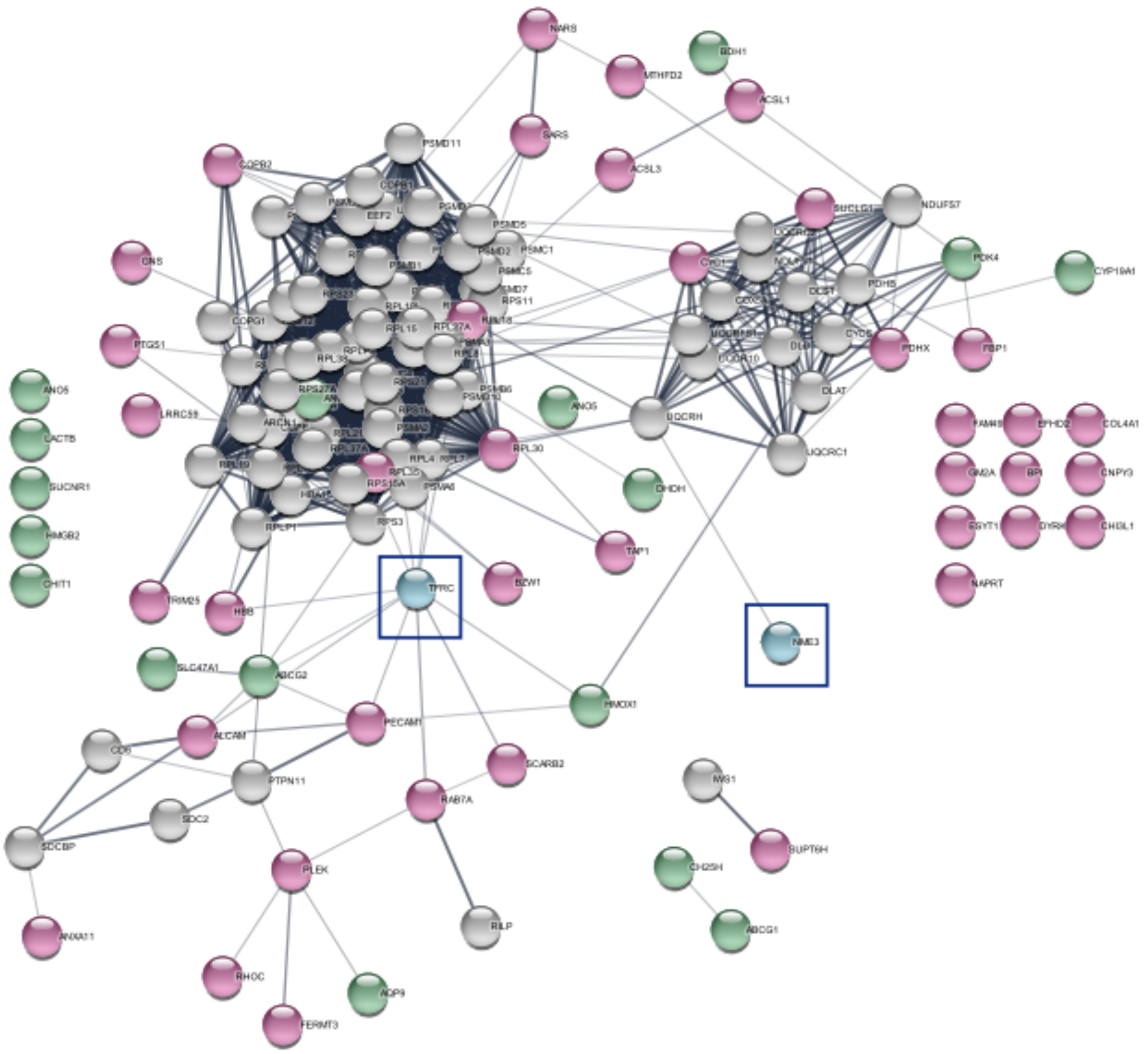

Figure S1. Enriched STRING network of differentially expressed proteins and transcripts. 60 combined proteins/transcripts from proteomics and transcriptomics obtained via STRING software[31] after progressive enrichment processes. [STRING basic settings – Network type: full STRING network (the edges indicate both functional and physical protein associations), meaning of network edges: confidence (line thickness indicates the strength of data support), active interaction sources: experiments, databases, co-expression, neighbourhood, gene fusion, co-occurrence), minimum required interaction score: medium confidence (0.400), max number of interaction to show: 1st shell (no more than 10 interactors), 2nd shell (custom value, max interactors: 60). Network stats – number of nodes: 130, number of edges: 1525, average node degree: 23.5, avg. local clustering coefficient: 0.635, expected number of edges: 552, PPI enrichment p-value: $< 1.0 \cdot 10^{-16}$]. Purple circles represent the differentially expressed proteins (DEP) from the proteomic experiments. Green circles represent the proteins corresponding to the differentially expressed transcripts (DET). Grey circles represent the proteins emerging from the sequential enrichment process from the initial 60. In light blue, NDK3 (NME1) and TRFC.

References:

[1] *García-Hernández, R.; Manzano, J. I.; Perea-Martínez, A.; Gamarro, F. New Insights on Drug-Resistant Clinical Isolates of Leishmania Infantum-Infected Human Macrophages as Determined by Comparative Transcriptome Analyses. OMICS* ***2022****, 26 (3), 165–177.* https://doi.org/10.1089/omi.2021.0185.

[2] Perea-Martínez, A.; García-Hernández, R.; Manzano, J. I.; Gamarro, F. Transcriptomic Analysis in Human Macrophages Infected with Therapeutic Failure Clinical Isolates of Leishmania Infantum. *ACS Infect. Dis.* **2022**, 8 (4), 800–810. https://doi.org/10.1021/acsinfecdis.1c00513.
